# Fish gut-liver immunity during homeostasis or inflammation revealed by integrative transcriptome and proteome studies

**DOI:** 10.1038/srep36048

**Published:** 2016-11-03

**Authors:** Nan Wu, Yu-Long Song, Bei Wang, Xiang-Yang Zhang, Xu-Jie Zhang, Ya-Li Wang, Ying-Yin Cheng, Dan-Dan Chen, Xiao-Qin Xia, Yi-Shan Lu, Yong-An Zhang

**Affiliations:** 1 Institute of Hydrobiology, Chinese Academy of Sciences, Wuhan 430072, China; 2 Demorgen Bioinformation Technology Co. Ltd, Wuhan 430072, China; 3 Fisheries College, Guangdong Ocean University, Zhanjiang 524088, China; 4 University of Chinese Academy of Sciences, Beijing 100049, China; 5 College of Fisheries and Life Science, Shanghai Ocean University, Shanghai 201306, China; 6 State Key Laboratory of Freshwater Ecology and Biotechnology, Wuhan 430072, China

## Abstract

The gut-associated lymphoid tissue, connected with liver *via* bile and blood, constructs a local immune environment of both defense and tolerance. The gut-liver immunity has been well-studied in mammals, yet in fish remains largely unknown, even though enteritis as well as liver and gallbladder syndrome emerged as a limitation in aquaculture. In this study, we performed integrative bioinformatic analysis for both transcriptomic (gut and liver) and proteomic (intestinal mucus and bile) data, in both healthy and infected tilapias. We found more categories of immune transcripts in gut than liver, as well as more adaptive immune in gut meanwhile more innate in liver. Interestingly reduced differential immune transcripts between gut and liver upon inflammation were also revealed. In addition, more immune proteins in bile than intestinal mucus were identified. And bile probably providing immune effectors to intestinal mucus upon inflammation was deduced. Specifically, many key immune transcripts in gut or liver as well as key immune proteins in mucus or bile were demonstrated. Accordingly, we proposed a hypothesized profile of fish gut-liver immunity, during either homeostasis or inflammation. Current data suggested that fish gut and liver may collaborate immunologically while keep homeostasis using own strategies, including potential unique mechanisms.

As the important constituent of the mucosal immune system, the gut-associated lymphoid tissue (GALT) constructs a local immune environment of both defensive and tolerant. The gastrointestinal tract is the main portals of pathogen entry both in mammals^1^ and lower vertebrates, such as fish^2^. And the liver, as the adjacent linked organ to GALT, contributes to immune surveillance^3^. Recently the liver has been clearly put forward as a central immunological organ with a high exposure to circulating antigens and endotoxins from the gut microbiota, particularly enriched for innate immune cells^4^. And disregulation of gut-liver immunity has been involved in many gut-liver diseases^3^. However, to date, in lower vertebrates, there are few reports regarding to gut-liver immunity. Fish gut-liver immunity remains largely unknown, even though enteritis as well as liver and gallbladder syndrome gradually emerged as a limitation in aquaculture, such as vegetable-containing meal induced inflammation of gut and liver^5^.

In mammals, the intestinal mucosal surface forms a biophysical barrier, and the mucus may enhance the homeostasis by delivering immunoregulatory signals^6^. The intestinal epithelial cells (IECs) can secret conditioning cytokines to prime intestinal dendritic cells for T cell response, meanwhile produce factors influencing on local IgA response^7^. In the lamina propria (LP), beneath IECs, both dendritic cells and macrophages have specific adaptations promoting tolerance, for that regulatory T cells (Tregs) and IgA^+^ B cells are induced as the main population, during homeostasis. However, upon shifting to inflammation, Th1 and Th17 responses are induced. Meanwhile, in liver, the inflammatory activation of hepatic stellate and Kupffer cells results in the chemokine-mediated infiltration of neutrophils, monocytes, NK and NKT cells^4^. The ultimate outcome of the intrahepatic immune response depends on the functional diversity of macrophages and dendritic cells, but also on the balance between pro- and anti-inflammatory T-cell populations^4^. Liver immune homeostasis can be rapidly restored through the apoptosis of CD8^+^ T cells after pro-inflammatory stimulation^8^. Meanwhile the homeostasis of CD4^+^ T cells, especially the balance between Treg and Th17 cells is important for liver health^9^. On the other hand, enterohepatic circulation of bile and blood carry products of digestion, along with antigens and microbial products^10^, as well as immune molecules and even lymphocytes^11^. And bile containing immune molecules, such as cytokines, chemokines, and antibodies, can modulate intestinal immunity to some extent^12^.

Fish GALT has recently become the subject of unprecedented research^13^. Fish intestine, especially the posterior segment, is immunologically active and armored with various immune cell types, including B cells, macrophages, granulocytes, and T cells^14^,^15^. The putative functions of T cells and antigen uptake mechanisms at fish gut mucosal surfaces were revealed preliminarily^16^. And intestinal natural and specific cell-mediated cytotoxicity was found in common carp and European seabass^16^. While, in fish liver, the exposure of liver cells to blood antigens, or to microbial products from the intestine, could also result in a distinctive local immune environment^17^. The fish intrahepatic immune cells (IHICs), responding upon challenge, were proved to exist in trout^18^. And morphological evidence for the presence of fish Kupffer cells has been provided^18^. Recently, zebrafish SIGIRR, a negative regulator for Toll/IL-1R, has been found essential to establish liver homeostasis under inflammatory stimuli^19^.

Furthermore, genomic, transcriptomic and proteomic studies shine lights on fish gut-liver immunity. At genomic level, the fact that most components associated with T lymphocyte function have been identified in fish suggested similar functionalities for fish and mammalian T lymphocytes in GALT^14^. At transcriptomic level, in fish liver, upon parasite infection, immune related KEGG pathways, including toll-like receptors, complement and coagulation cascades, and chemokine signaling pathways, were activated^20^; while exposed to environmental toxicants, many immune-related pathways, including natural killer cell mediated cytotoxicity, T-cell signal transduction and T-cell receptor signaling pathway, as well as B-cell related pathways, were repressed^21^; during soybean-induced enteritis, TNF signaling pathway, NOD-like receptor interaction, NF-κB signaling pathway, cytosolic DNA sensing pathway, Jak-STAT signaling pathway, cytokine-cytokine receptor interaction and T-cell receptor signaling pathway were revealed in gut, meanwhile complement cascade was found to be of most important in liver^5^. Proteomic study of rainbow trout intestinal epithelia reflected that short-term starvation could change IECs at protein level^22^; and substituting fish meal in fish feed by alternative sources of protein could alter fish gut proteome, including innate immune proteins, such as C3 and transferrin^23^. However, the detail information of fish gut-liver immunity still remains unclear, thus the systemic and comprehensive analysis is in urgent need.

In addition, the most consistent pathological changes in tilapia infected by *Streptococcus agalactiae* were marked congestion of internal organs, particularly the liver, spleen and kidneys^24^, and interestingly the main entry site of *S. agalactiae* in tilapia was the gastrointestinal epithelium^2^, thus fish intestinal mucus seems to play an important defensive role. Therefore, tilapias infected by *S. agalactiae* were used as the animal model to study the gut-liver immunity in fish. In this study, the gut and liver tissues from both healthy and infected fish were applied for digital gene expression (DGE) profiling, meanwhile intestinal mucus and bile were assessed for by isobaric tags for relative and absolute quantitation (iTRAQ) as a supplementary of body fluid to understand the link between gut and liver, in order to demonstrate the fish gut-liver immunity. The comparison was done in data of both inter-tissue/fluid (gut vs liver, and mucus vs bile) and intra-tissue/fluid (gut, liver, mucus or bile). Besides analysis by GO, KEGG, *etc.et,* many key immune transcripts in gut or liver as well as key immune proteins in mucus or bile were revealed, using current constructed fish immune gene library. Accordingly a hypothesized portrait of fish gut-liver immunity, including potential unique mechanisms, at both steady and inflammational status, was firstly proposed.

## Results

### Analysis of differential transcripts in gut or liver

**Figure 1:**
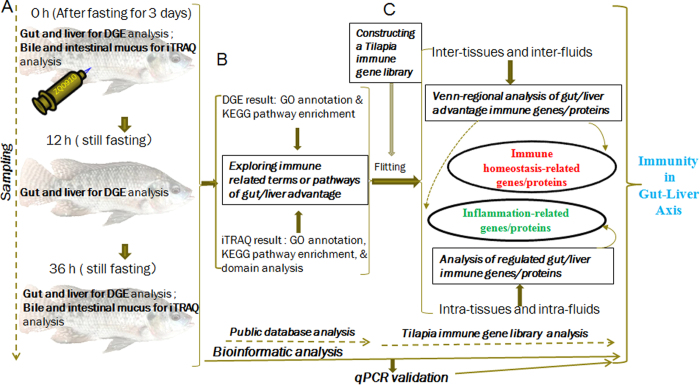
Strategy for identification of regulated immune genes in fish gut and liver by integrative analysis of both DGE transcriptomic and iTRAQ-based quantitative proteomic data. (**A**) Tilapias were infected intraperitoneally with live *S. agalactiae*, and then sampling for DGE profiling of gut or liver was done at 0 h (just before challenge), together with 12 h and 36 h post challenge, meanwhile sampling for iTRAQ analysis of intestinal mucus or bile was done at 0 h and 36 h, from both healthy and infected fish; (**B**) Immune related annotation was found out *via* bioinformation analysis of both DGE and iTRAQ results using public database (including GO, KEGG, and InterPro); (**C**) On one hand, the comparison was done in both inter-tissue/fluid data (gut vs liver, or intestinal mucus vs bile) and intra-tissue/fluid data (gut, liver, mucus or bile), then Venn-regional analysis was applied to screened out immune homeostasis-related transcripts and proteins; on the other hand, regulated gut-liver immune transcripts or proteins were revealed by intra-tissue (same tissue at different time-points) or intra-fluid (same fluid at different time-points) comparison.

For DGE results, the raw, clean and mapped reads for each sample were listed in Table S1. In order to carry out immune function analysis for tissue advantage, the main differential transcripts between gut and liver were divided into two main populations (gut advantage (>2 fold change in gut) and liver advantage (>2 fold change in liver)). After sampling (Fig. 1A) for DGE profiling and public database analysis, matched immune related GO terms and KEGG pathways were elucidated (Fig. 1B, Table 1, and Fig. S1). The common terms or pathways were about immune response, immune system process, integrin complex, cytokine activity, phagosome, and cell adhesion molecules, while those specifically for gut advantage were about intestinal IgA as well as antigen processing and presentation. And it is worth mentioning that the gut advantage transcripts were much highly involved in the immune related part of the pathway “cell adhesion molecules”, including molecular interfaces for T cell receptor signaling pathway, tight junction, leukocyte transendothelial migration, and *et al*. (Fig. S2A–C). Notably, the leukocyte transendothelial migration was suggested very highly gut advantage just at 0 h (Fig. S2A). Among different transcripts between gut and liver, the lymphocyte migration related chemokines (in the pathway “cytokine-cytokine receptor interaction”, Fig. S2D–F) and integrins (in the pathway “ECM-receptor interaction”, Fig. S2G–I) were found with most significantly variation comparing data of 0 h, 12 h and 36 h. In addition, the fact that the highest *R*^2^ was between 0 h and 36 h (Fig. S3) in the result of correlation analysis among the differential transcripts of 3 time-points, indicated the maximum similarity between data of 0 h and 36 h, in another word imply recover of certain degree at 36 h. Later, in qPCR validation result, among 26 reactions of 14 genes, fold-changes between DGE and qPCR results correlated well (Fig. S4 and Table S2).

### Analysis of differential proteins in mucus or bile

For iTRAQ results, the annotation and detailed information of all identified proteins as well as summary for each comparison group were reflected by Table S3. In order to elucidate mucus or bile advantage proteins (>2 fold change), sampling of iTRAQ analysis (Fig. 1A) and selecting immune related GO, KEGG, and InterPro (Fig. 1B) were carried out. The common terms or pathways were “immune system process” as well as “complement and coagulation cascades”, while the specific one for mucus advantage is “antigen processing and presentation” (Table 1 and Fig. S5A,B). For domains, more immune related predicted ones in bile than mucus, meanwhile many common immune-related ones between bile and mucus, such as complement components, acute phase proteins (APPs), immunoglobulin, *et al*., were revealed (Table 2 and Fig. S5C).

### Classification of gut or liver advantage transcripts by the tilapia immune gene library

**Figure 2:**
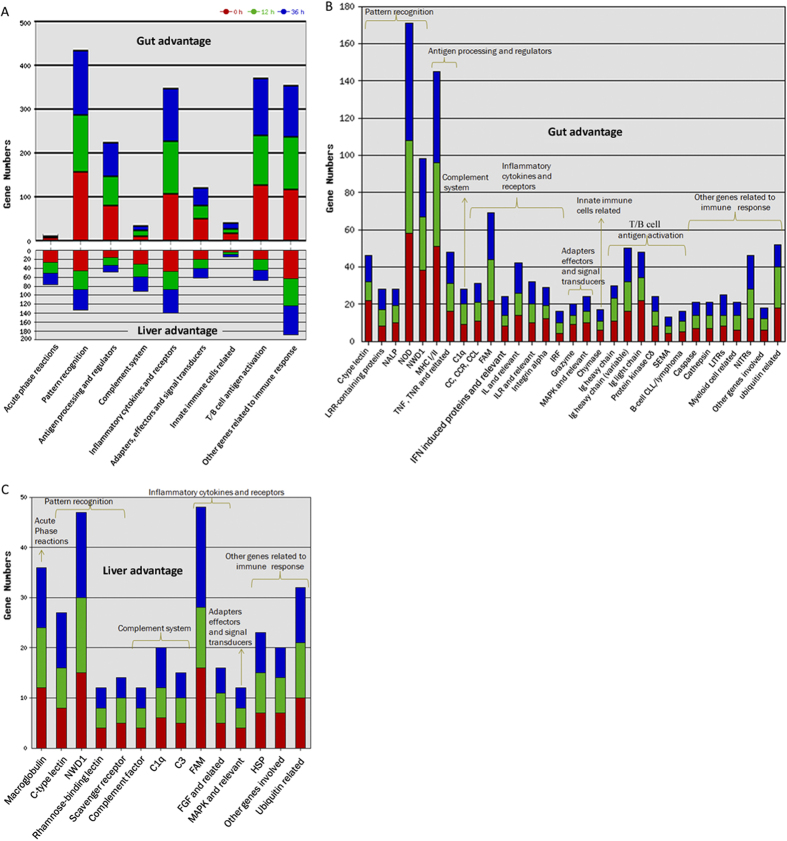
Classification of fish immune transcripts of gut or liver advantage by two levels of the tilapia immune gene library. (**A**) Major immune processes (at the first level) involved in gut and liver; (**B**) Immune gene categories (at the second level) involved in gut; (**C**) Immune gene categories (at the second level) involved in liver. The gut advantage transcripts were significantly involved in “pattern recognition” (gut vs liver, 156:45 at 0 h, 128:42 at 12 h, and 148:46 at 36 h), “antigen processing and regulators” (77:15 at 0 h, 69:18 at 12 h, and 76:15 at 36 h), “inflammatory cytokines and receptors” (107: 46 at 0 h, 121: 41 at 12 h, and 121: 53 at 36 h), “adapters, effectors and signal transducers” (49:20 at 0 h, 29:20 at 12 h, and 42:21 at 36 h), “innate immune cells related” (15:4 at 0 h, 11:5 at 12 h, and 13:5 at 36 h), “T/B cell antigen activation” (126:20 at 0 h, 113:24 at 12 h, and 130:23 at 36 h), and “other genes related to immune cell response” (114:63 at 0 h, 122:60 at 12 h, and 115:65 at 36 h), while compared to gut, liver advantage transcripts were significantly involved in “acute phase reactions” (liver vs gut, 26:4 at 0 h, 25:3 at 12 h, and 25:3 at 36 h) and “complement system” (30:10 at 0 h, 29:12 at 12 h, and 32:9 at 36 h).

We used current construct tilapia immune gene library (Table 3 and detailed in Table S4) to classify the revealed transcripts and proteins. The total No. of immune transcripts was much greater for gut advantage than liver advantage. With respect to immune process (Fig. 2A), gut advantage transcripts were significantly involved in “pattern recognition”, “antigen processing and regulators”, “inflammatory cytokines and receptors”, “adapters, effectors and signal transducers”, “innate immune cells related”, “T/B cell antigen activation”, and “other genes related to immune cell response”, while liver advantage transcripts were significantly involved in “acute phase reactions” and “complement system”. In detail, the most enriched (>40) immune gene categories among gut advantage transcripts (Fig. 2B) were NOD, MHC I/II, NWD1, FAM, Ig heavy chain, Ig light chain, C-type lectin, ubiquitin related, NITRs, TNF, TNR, and related, as well as IL and relevant. Whereas, among liver advantage transcripts (Fig. 2C), the most enriched (>20) immune gene categories were FAM, NWD1, macroglobulin, ubiquitin related, C-type lectin, HSP, C1q, as well as other genes involved. Furthermore, the highest correlation coefficient of differential transcripts emerged upon comparing data of 0 h and 36 h (Fig. S3) indicated that the transcription of immune genes was more close to homeostasis at 36 h rather than 12 h. Additionally the No. for specific gene categories was shown in Table S5.

### Classification of mucus or bile advantage proteins by the tilapia immune gene library

**Figure 3:**
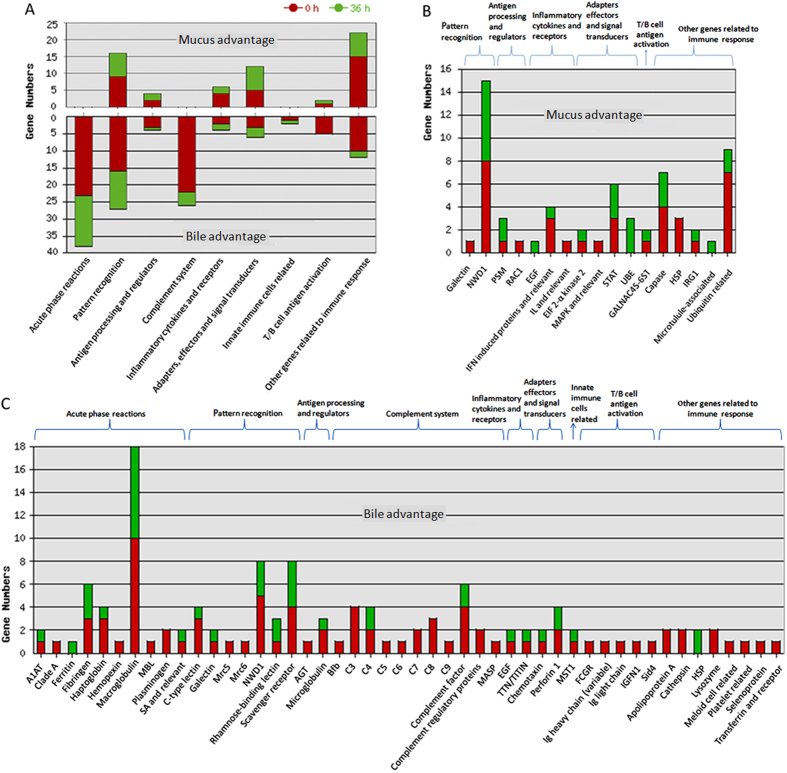
Classification of fish immune proteins of intestinal mucus or bile advantage by two levels of the tilapia immune gene library. (**A**) Major immune processes (at the first level) involved in intestinal mucus and bile; (**B**) Immune gene categories (at the second level) in intestinal mucus; (**C**) Immune gene categories (at the second level) in bile. Mucus specific advantage proteins were mainly involved in “inflammatory cytokines and receptors” (mucus vs bile, 4:2 at 0 h, and 2:2 at 36 h) and “other genes related to immune cell response” (15:10 at 0 h, and 7:2 at 36 h). While, the bile specific ones were mainly involved in “acute phase reactions” (bile vs mucus, 23:0 at 0 h, and 15:0 at 36 h), “complement system” (22:0 at 0 h, and 4:0 at 36 h), “innate immune cells related” (1:0 at 0 h, and 1:0 at 36 h). The common involved immune processes were mainly in “pattern recognition” (bile vs mucus, 16:9 at 0 h, and 11:7 at 36 h) and T/B cell antigen activation (5:1 at 0 h, and 0:1 at 36 h).

Total No. of immune proteins was much greater for bile advantage than mucus advantage. With respect to immune process (Fig. 3A), mucus specific advantage proteins were mainly involved in “inflammatory cytokines and receptors” and “other genes related to immune cell response”; and bile specific ones were mainly involved in “acute phase reactions”, “complement system”, and “innate immune cells related”. The common involved immune processes between mucus and bile were mainly in “pattern recognition” and “T/B cell antigen activation”. In detail, the mostly enriched (>4) immune gene categories among mucus advantage proteins (Fig. 3B) were NWD1, ubiquitin related, caspase, STAT, as well as IFN induced proteins and relevant. Whereas, among bile advantage proteins (Fig. 3C), the mostly enriched (>4) immune gene categories were macroglobulin, NWD1, scavenger receptor, complement factor, fibrinogen, haptoglobulin, C-type lectin, C3, C4, and perforin 1. And higher correlation coefficient of differential immune proteins rather than that of total ones (Fig. S3) suggested conserved immune functional division between bile and mucus. The No. for specific gene categories was shown in Table S6.

### State-dependent analysis of differential immune transcripts between gut and liver

**Figure 4:**
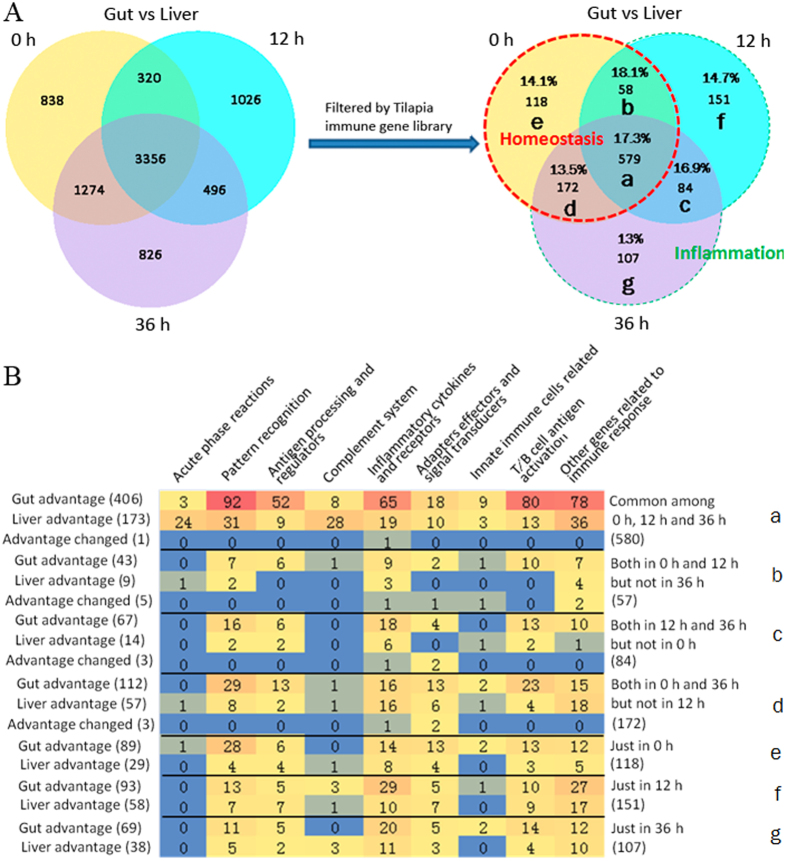
Venn-regional analysis of gut or liver advantage transcripts. (**A**) Venn analysis among data of 0 h, 12 h, and 36 h, for both the total and immune differential transcripts between gut and liver, and the percentage of immune transcripts vs total transcripts was labeled in each region. The total No. of differentially expressed genes between gut and liver was 5788, 4702 and 5952 at 0 h, 12 h and 36 h respectively, and after filtered by the immune gene library the numbers decreased to 927, 872, and 942. And the dashed red lined regions, including region a, b, e and d, indicated data at 0 h, in another word in homeostasis status, whereas the dashed green lined regions (f, c and g) specifically indicated data upon inflammational states (12 h and 36 h). (**B**) Transcripts No. involved in major immune processes for each region. For gut advantage transcripts, in regions a, b and c, most of them were involved in “pattern recognition”, “T/B cell activation”, “other genes related to immune response”, “inflammatory cytokines and receptors”, as well as “antigen processing and regulators”; in regions d and e, except for the above mentioned, most of them were also involved in “adapters, effectors and signal transducers”; in regions f and g, most of them were involved in “pattern recognition”, “inflammatory cytokines and receptors”, “T/B cell antigen activation”, as well as “other genes related to immune response”. On the other hand, for liver advantage ones, in region a, most were involved in “other genes related to immune response”, “complement system”, “pattern recognition”, as well as “acute phase reactions”. In regions d and g, most were involved in “other genes related to immune response”, “inflammatory cytokines and receptors”, as well as “pattern recognition”, while in region f, also in “antigen processing and regulators”, as well as “T/B cell activation”, except for the above mentioned. In other regions (b, c and e), the liver advantage genes were much fewer.

In Venn diagram of differential immune transcripts between gut and liver among 0 h, 12 h and 36 h (Fig. 4A), the percentages (around 15%) of immune transcripts in all differential regions, with relatively higher percentages in regions related to inflammation (especially at 12 h), indicated the basic defense at steady state as well as more intensive defense during inflammation. In all regions, No. of gut advantage genes was always greater than those of liver (Fig. 4B). All the immune transcripts at 0 h might be related to immune homeostasis. In detail, the immune transcripts in region e might be related to immune homeostasis, in region b and d to regulation of immune homeostasis, whereas in region a to the basic functional division between fish gut and liver. On the other hand, the immune transcripts in other regions (c, f, and g) may be certainly related to different developing stages of inflammation. Then regional components at the level of immune process according to the tilapia immune gene library were analyzed (Fig. 4B). Among Venn-regional transcripts (detailed in Table S7), in homeostasis related regions (a, b, d and e), MHC II, immunoglobulin, SOCSs, LITRs, IL1R, Foxp3, CD73, C-type lectin, chymas, MCP, *et al*. were found for gut advantage, and APPs, perforin, TGF, complement components, antimicrobial peptides, NCAM1, scavenger receptors, c-type lectin, CD73, IL1R, IL11R, IL13R, *et al*. were revealed for liver advantage, in specifical regions. On the other hand, in inflammation related regions (f, c and g), IFN-γ and IFN induced proteins, immunoglobulin heavy chain, IL-1β, CCR6, CD3, CD8, F-type lectin, IL-17D, CD93, CD244, *et al*. were found for gut advantage, and MMD, IL-17C, RGS5b, C1q, *et al*. were revealed for liver advantage, in specifical regions. In addition, the transcripts of changed tissue advantage, such as IL10, IL11, NFKBI, IpLITRs, *et al*., could be the most significantly regulated upon inflammation and coincident with results of intra-tissue analysis.

### State-dependent analysis of differential immune proteins between mucus and bile

**Figure 5:**
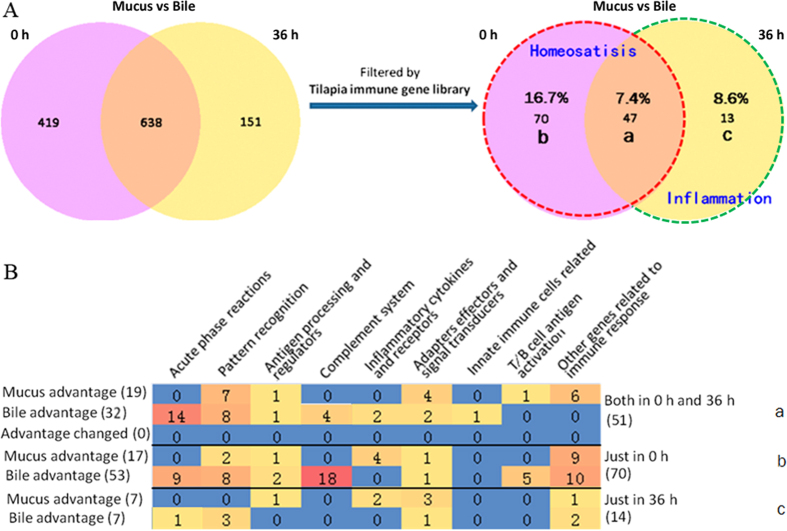
Venn-regional analysis of intestinal mucus or bile advantage proteins. (**A**) Venn analysis between data of steady (0 h) and inflammational (36 h) for both the total and immune differential proteins between intestinal mucus and bile, and the percentage of immune proteins vs total proteins was labeled in each region. The total No. of differentially expressed genes between intestinal mucus and bile was 1057 and 789 at 0 h and 36 h respectively, and after filtered by immune gene library the numbers decreased to 117 and 60. The dashed red lined regions, including region a and b, indicated data at 0 h, in another word in homeostasis status, whereas the dashed green lined region (c) specifically indicated data upon inflammation (36 h). (**B**) Protein No. involved in major immune processes for each region. In region a, the most enriched mucus advantage proteins were involved in “pattern recognition”, “adapters, effectors and signal transducers”, as well as “other proteins related to immune response”; meanwhile, for bile advantage ones, in “acute phase reactions”, “pattern recognition”, and “complement system”. In region b, the most enriched mucus advantage ones were involved in “other proteins related to immune response” and “inflammatory cytokines and receptors”; meanwhile, for bile advantage ones, in “complement system”, “other proteins related to immune response”, “acute phase reactions”, “pattern recognition”, and “T/B cell antigen activation”. In region c, there were much fewer immune proteins, with the most enriched mucus advantage ones in “adapters, effectors and signal transducers” as well as in “pattern recognition” for bile.

Since that fish transferred to indoor farming system was starving for 3 days before sampling, at 0 h swelling bile bladder was found (data not shown). All the immune proteins at 0 h might be related to maintain immune homeostasis in fish gut and liver. In detail, the proteins only at 0 h (region b in Fig. 5A) might be related to steady status, whereas the common ones (region a in Fig. 5A) to basic division. At 36 h, flabby bile bladder (data not shown) suggested release of bile to intestinal mucus, and also echoed with dramatically decreased differential proteins (13 ones only at 36 h, in region c of Fig. 5A) synchronously. Then regional compositions at the level of immune process according to the tilapia immune gene library were analyzed (Fig. 5B). Among Venn-regional proteins (detailed in Table S8), in homeostasis related regions (a & b), galectin, STAT3, STAT5, *et al*. were found for mucus advantage, and complement inhibitors, APPs, lysozyme, *et al*. were revealed for bile advantage, in specifical regions. Meanwhile, in inflammation related region c, UBE, EGF, IFN induced protein, *et al*. were found for mucus advantage, and perforins, HSP, RBL, *et al*. were revealed for bile advantage. In addition, there was no protein revealed for changed advantage.

### Overlap between liver and bile advantage immune genes

**Figure 6:**
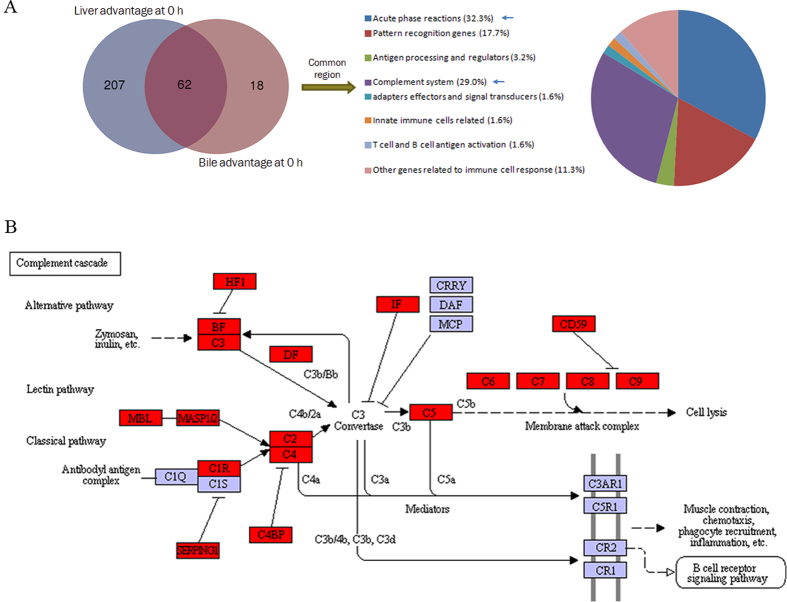
Analysis of common genes between liver advantage transcripts and bile advantage proteins. (**A**) Venn analysis between genes of both liver advantage transcripts and bile advantage proteins, with percentages of common components. (**B**) The KEGG pathway graphic of complement cascades in bile advantage proteins at steady state (0 h). Three complement activating pathways as well as inhibitory factors were revealed.

The 62 common immune genes, between liver and bile advantage ones (Table S9), were mainly involved in “acute phase reaction”, “complement system”, “pattern recognition genes”, and “other genes related to immune cell response” (Fig. 6A). Notably, both in results of KEGG and tilapia immune gene library analysis, the complement system (Fig. 6B), including classic, alternative, and MBL pathways, was significantly matched both in liver and bile. Meanwhile, inhibitory factors of complement system, such as factor H1 (HF1, or CFH), serpin peptidase inhibitor, clade C (SERPINC1), C4b-binding protein (C4BP), factor D (IF, or CFI), and CD59, were also found at steady state (Fig. 6B). Also many innate effectors, as potential antimicrobial peptides (cathepsin La) and lectins (rhamnose-bingding lectin (RBL) and c-type lectin), were revealed (Table S9).

### Regulated immune transcripts in gut or liver comparing steady and inflammatory states

Among regulated immune transcripts in the tilapias’ gut or liver (listed in Table 4 and detailed in Table S10), according to the inflammation process, in both gut and liver (36 h vs 0 h, and 12 h vs 0 h), down-regulated ones might be related to homeostasis, whereas up-regulated ones to inflammation. For differential transcripts between 36 h and 12 h in gut, all might be related to inflammation, whereas in liver there were overlaps, between up-regulated (36 h vs 12 h) and down-regulated (12 h vs 0 h) and also between down-regulated (36 h vs 12 h) and up-regulated (12 h vs 0 h) ones, which may contain the key regulators related to maintain steady state and turnover from steady to inflammational status, respectively. The up-regulated (12 h vs 0 h) and down-regulated (36 h vs 12 h) liver transcripts were found of most abundant. And also some fish specific genes were also revealed, such as up-regulated (36 h vs 0 h) intestinal fish-egg lection (Table 4).

### Regulated immune proteins in mucus or bile comparing steady and inflammatory states

In contrast to many regulated immune transcripts in tilapias’ gut and liver, the significantly regulated immune proteins in fluids in gut and liver, including mucus and bile, were much fewer. Among regulated proteins in the tilapias’ mucus or bile (listed in Table 5 and detailed in Table S11) during inflammatory process, in both mucus and bile (36 h vs 0 h) down-regulated ones were related to homeostasis, whereas up-regulated immune ones to inflammation. In addition, up-regulated proteins in mucus (36 h vs 0 h) might be related to the incorporated bile components at 36 h.

## Discussion

The fish gut-liver immunity is largely unknown and related studies are still scattered, thus systemic analysis is in need. In this study, integrative bioinformatic analysis of both transcriptomic and proteomic data of both healthy and infected tilapias revealed many key immune genes in fish gut and liver, thus firstly elucidated a sophisticated profile of fish gut-liver immunity during both homeostasis and inflammation.

The less differences and higher correlation coefficient of immune transcripts between fish gut and liver upon inflammation suggested their enhanced synchronization although with individual responsibility. Among gut advantage transcripts, those involved in “pattern recognition” and “T/B cell antigen activation” were mostly enriched. The current revealed transcripts for pattern recognition genes in fish gut was in line with the essential role of pattern recognition receptors, widely expressed on innate immune cells and IECs, for the recognition and clearance of commensal and pathogenic microflora^25^. Meanwhile, gut advantage transcripts for “T/B cell antigen activation” echoed with the matched KEGG pathways. The fact that more intestinal Ig light chain transcripts at steady state might indicate T cell-independent IgT response, likely involved in cross-reacting with commensal bacteria, for that mammalian Abs from T cell-independent IgA^+^ B cells often use Ig lambda light chain, and were polyreactive^26^. So as to liver, transcripts involved in “acute phase reactions” and “complement system” were consistent with findings in trout’s inflammatory liver^7^. Fish hepatocytes are also the prime source of APPs^27^. And among the liver advantage complement genes, the greatest No. of C1q indicated highly involving of classical complement pathway, meanwhile existence of both the negative (CFH and CFI^28^) and positive (CFP^29^) regulators for alternative pathway indicated that the alternative complement pathway could be of great importance in fish liver^30^,^31^.

Further, the immune transcripts revealed by Venn-regional analysis also shined lights on key responders or regulators of fish gut-liver immunity. The percentages of immune genes in Venn regions indicated the basic defense at steady state as well as more intensive defense during inflammation. The regions a, b, d and e in DGE diagram (Fig. 4A) may possibly be related to homeostasis. The much more immune transcripts in gut in region a, involving in phagocytosis (eg. cathepsins^30^), antigen presentation and cytokine signaling, and finally T/B cell response, implied much intense and adaptive intestinal immune reaction. While in liver, the innate transcripts, mainly involving in “acute phase reactions” and “complement system”, may play an important role. Among gut advantage ones, many MHC (mainly II) and immunoglobulin (IgM and the other, maybe IgT) transcripts might imply existing of fish MHC II-expressed intestinal innate lymphocytes (ILCs), since that such population may limit expansion of pathological CD4^+^ T cell and therefore induce IgA production in mammalian intestine^32^. And many immunoregulatory factors were also found in gut advantage transcripts, such as SOCSs (suppressor of cytokine signaling proteins, may inhibit the activity of JAKs and STATs^33^) in region a, LITRs (Leukocyte immune-type receptors, might inhibit cellular cytotoxicity^34^) in region d, IL-1R (may neutralize IL-1) in region d, as well as Foxp3 in region a and CD73 in region d (might be the key transcription factor^35^,^36^ and effecter^37^,^38^,^39^ of Treg respectively). Also intestinal c-type lectins in regions a, d and e suggested their positive relation to maintain immune homeostasis^40^, which was coincident with that mammalian CLRs (C-type lectin receptors) on macrophages or dendritic cells (DCs) are involved in IL-10 production and Treg maturation^6^,^41^. Moreover, intestinal chymas in regions a and b, as well as mast cell protease (MCP) in region e, indicated involvement of mast cells (MCs) in keeping gut homeostasis, coincident with that mammalian MCs prefer to localize in mucosal healing areas^42^.

On the other hand, among liver advantage transcripts, in region a, APPs, perforin, TGF, complement components, and antimicrobial peptides were found conserved in fish liver as in mammals; NK cell-specific makers, such as NCAM1 and many scavenger receptors^30^,^43^, suggested exist of NK-like cells; c-type lectin might be related to Kupffer cell polarization for improving homeostasis^44^. In region b, Treg’s effector CD73 suggested possible existence of Tregs in fish liver. In addition, IL-1R in region d, IL-11R in region a, and IL-13R in region e suggested inhibition of inflammatory cytokines. Another fact that more integrin transcripts existed in gut than liver, and was found particularly in homeostasis related regions, suggested homeostasis related migration of lymphocytes^45^ and dysregulation of lymphocytes migration upon inflammation^27^.

In the rest regions (f, c and g, inflammation related), for gut advantage transcripts, in region f, IFN-γ and IFN induced proteins implied Th1 response^46^; and the immunoglobulin heavy chain (μ and another one) transcripts suggested coexistence of IgM and probably IgT^47^,^48^. In region c, proinflammatory factors (IL-1β and CCR6) and T cell markers (CD3 and CD8) indicated CTL responses occurred in the inflammatory gut^49^; and F-type lectin was in line with other reports of fish inflammation^50^,^51^. In region g, IL-17D might indicate Th17 response, since that IL-17D triggers secretion of IL-6 (the typical Th17 type cytokine)^52^; CD93 (C1qR) could facilitate phagocytosis^53^; and CD244 could probably mediate activation of NK-like cell^54^. On the other hand, for liver advantage transcripts, in region c, MMD (monocyte to macrophage differentiation-associated protein) indicated expansion of Kupffer cells (KCs) in liver^55^. In region f, IL-17C suggested promotion of Th17 response^56^; RGS5b (regulator of G-protein signaling 5b) indicated roles of hepatic satellite cells upon inflammation^57^. In addition, C1q in regions f and g could facilitate complement activation and phagocytosis^58^.

Moreover, the regulated transcripts in gut or liver, comparing steady and inflammatory status, provided another clue to assess genes related to both homeostasis and inflammation in fish gut-liver immunity. Though the regulated transcripts in gut or liver echoed with the results of Venn regional analysis partially, they added many important components, especially for liver since that the ip. injection, which could also induce immune response of fish gut and liver^16^, certainly caused more stress on liver^59^. Among the significantly pro-inflammatory up-regulated transcripts in liver (12 h vs 0 h), IL-10 might dampen the inflammatory response^60^; CEBPβ (CCAAT element binding protein β) and RBPJ (recombination signal binding protein for immunoglobulin kappa J region, found in both liver advantage transcripts of region d and regulated transcripts in liver, including up-regulated 12 h vs 0 h and down-regulated 36 h vs 12 h), indicated activation of KCs^61^,^62^. And, NFIL3 (nuclear factor, interleukin 3 regulated), up-regulated in both gut and liver (12 h vs 0 h), suggested the pro-inflammatory expansion of ILCs^63^ (including helper ILCs and NK-like cells^64^). While, down-regulated NK-lysin (12 h vs 0 h), implied the important role of NK-like cells in fish liver even in steady state^65^. Meanwhile, the inflammation up-regulated fish-egg lectin in gut was coincident with its ability for enhancing the phagocytosis of the bacteria by macrophages^66^ in zebrafish.

As important supplementary, the iTRAQ results of intestinal mucus and bile not only confirmed the DGE results of gut and liver but also shed new light on fish gut-liver immunity. In general, more immune proteins in bile than mucus could be found, and bile might provide immune molecules for mucus upon inflammation^67^. Among mucus advantage proteins, as confirmation, the fact that significantly down-regulated galectin upon inflammation, also was found in both mucus advantage proteins of region b and gut advantage transcripts in regions a and d, might indicate its immunomodulatory role in fish^6^,^68^. Whereas, as new findings, the fact that STAT3 and STAT5 in mucus advantage proteins of region a suggested the homeostasis mechanism of fish IECs, since that in mammalian IECs, STAT3 regulates intestinal homeostasis and mucosal wound healing^69^, and STAT5 promotes proliferation and regeneration to mitigate intestinal inflammation^70^. On the other hand, among bile advantage proteins, at steady state (region b) complement inhibitors and acute phase proteins were found for keeping homeostasis, and also lysozyme might trigger an opsonin of the complement system and phagocytic cells^31^. Upon inflammation (region c), other innate effecter, such as perforin and RBL, were also identified in bile.

And, the regulated proteins in either mucus or bile also provided essential information for fish gut-liver immunity at protein level. In mucus, C1q binding proteins, as the most significantly down-regulated category upon inflammation (36 h vs 0 h), might imply their ability in maintaining homeostasis because of their inhibitory role for complement pathway in mammals^71^. While, the up-regulated CFD (complement factor D) in mucus (36 h vs 0 h) suggested the activation and amplification of the alternative complement pathway at mucus surface^72^. On the other hand, in bile, CD59, down-regulated (36 h vs 0 h), suggested enhancing of membrane attack^73^. Additionally, LECT2 (leukocyte cell-derived chemotaxin 2) and RBL found in the up-regulated proteins of both mucus and bile upon inflammation imply their anti-microbial role.

**Figure 7:**
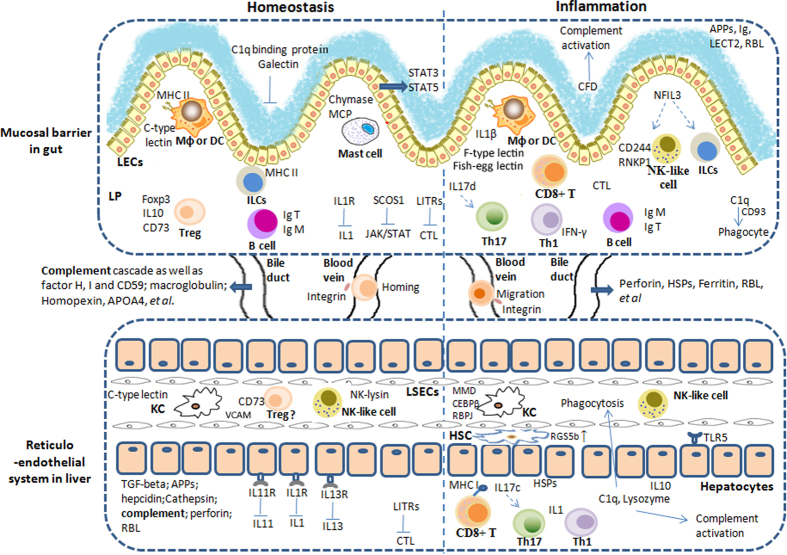
Hypothesized fish gut-liver immune mechanisms involved in either homeostasis or inflammation. The hypothesized portrait of fish gut-liver immunity was drawn accordingly to the discussion of current revealed immune genes in tilapias’ gut and liver. Besides immune components at both intestinal mucosal barrier and liver reticulo-endothelial system, intestinal mucus and bile also contain many immune modulators or activators. During immune homeostasis, on one hand, in gut, genes of possible immunomodulatory role, including innate immune molecules, such as galectin and c-type lectin, as well as regulatory T/B cell related ones, together with genes responsible immune-suppression, such as IL1R, were found with abundance. On the other hand, in liver, in addition to immunomodulatory and immune suppression genes, innate immune molecules, such as acute phase proteins, complement components and anti-microbial peptides, which could be delivered from bile to intestinal mucus, were found of great importance for basic function. In addition, molecules (chemokines and integrins, with some members different from mammals) related to migration of lymphocytes between gut and liver were also inferred particular at steady state (termed homing). While upon inflammation, in gut, the immune genes, responsible for immune activation, including both innate ones, such as fish-egg lection (fish-specific), CFD and C1q, and adaptive ones, mainly T cell response (CD8^+^T, Th1 and Th17) related, were prevailing. At the same time in liver, genes for activation of immune responses, mainly including innate immune molecules and cells, together with relative lower T cell response, were found. Innate immune factors could be also transported from bile to intestinal mucus upon inflammation. Genes labeled or related to immune cells in the diagram were all highlighted in Tables S6 and S7.

In conclusion, current data suggested that in fish intestinal immunity genes related to antigen recognition and presentation as well as activation of T/B lymphocytes were mostly involved, meanwhile the fish liver immunity possesses many factors in keeping immune homeostasis, and the innate immune (especially the complement system) plays an important role. So as to the immunity communication between fish gut and liver, we found that bile could provide immune proteins to the intestinal mucus both at homeostasis and upon inflammation, and immune proteins facilitating immune tolerance or activation did exist in fish intestinal mucus. Additionally, the probably migration of lymphocytes between gut and liver could be inferred from DGE result. Thus, current data suggested that fish gut and liver may collaborate immunologically while keep homeostasis using own strategies (Fig. 7), *via* immune molecules and cells, deduced from fish immune gene library analysis result as well as the KEGG pathway (Fig. S2A–C). Additionally the reticuloendothelial structure of fish liver was elucidated by our previously study^43^. Thus fish gut-liver immunity structurally consists of mucosal barrier in gut and reticuloendothelial system in liver, together with fluids (bile, blood, and mucus).

Yet, some particular clues for fish gut-liver immunity also emerged, such as the regulated chemokines and integrins possibly involved in lymphocyte migration and homing, for example, the finding that there was no integrin β7 revealed in tilapia, although mammalian integrin α4β7 facilitating migration for gut-homing of lymphocytes, indicated possible unique mechanism. And the inflammation up-regulated fish-egg lectin in fish intestinal barrier could also be special. Therefore some unique mechanisms for fish gut-liver immunity may exist. Although many interesting immune factors and clues for hypothesized immunity in fish gut and liver were revealed by current study, further efforts still should be made on elucidating gene function and involved mechanisms.

## Methods

### Fish, bacterial strain, challenge and sampling

The 30 adults (approximately 500 g) of GIFT strain Nile tilapia (*Oreochromis niloticus*) were collected from Xuwen inshore fishery, Guangdong Province, China. And the live *S. agalactiae* (strain ZQ0910, which was isolated from diseased fish in Guangdong Province, China^74^) was used to challenge tilapias. After adapting to the indoor circulating water system for 3 days, the tilapias were intraperitoneally injected with 0.1 ml concentrated ZQ0910 (5*10^8^ CFU/ml, diluted using normal saline). For DGE profiling, a series of liver or gut tissue samples from fish (about 3–5 fishes for each time-point) of different stages (0 h, or in another word just before challenge, as well as 12 h and 36 h post challenge). were dissected, for frozen immediately in liquid nitrogen, followed by storage at −80 °C until RNA extraction. And for iTRAQ analysis, bile or posterior intestinal mucus from 3–5 fishes was collected and low speed (at 500 × g for 10 min) centrifugated in order to remove contamination particles. The use of animals in this study was approved by the Animal Research and Ethics Committees of Institute of Hydrobiology, Chinese Academy of Sciences, and all experiments were conducted in accordance with the guidelines of the committees.

### RNA extraction, library preparation and sequencing

Tilapia tissues of either liver or posterior intestine, at steady or different inflammational stages, were collected and the total RNA was isolated from each sample using Tiangen RNA prep Pure Plant Kit (Tiangen Biomart, Beijing). 20 mg total RNA from each sample was sent to Novogene Bioinformatics Technology Co. Ltd (Beijing). RNA quality and quantity were determined by a Nano Photometer spectrophotometer (IMPLEN, CA, USA), a Qubit RNA Assay Kit in a Qubit 2.0 Flurometer (Life Technologies, CA, USA) and a Nano 6000 Assay Kit that was part of the Agilent Bioanalyzer 2100 system (Agilent Technologies, CA, USA). Among total of 10 mg RNA, 1 mg from each of the three samples, was used as the input material for the transcriptome library and 3 mg RNA per sample was used for the DGE library. Briefly, the mRNA was purified by poly-T oligo-attached magnetic beads and fragmented by divalent cations under elevated temperature in NEB Next First Strand Synthesis Reaction Buffer. Random hexamer primer and M-MuLV Reverse Transcriptase (RNase H) were used for first strand cDNA synthesis. Second strand cDNA synthesis was subsequently performed using DNA Polymerase I and RNase H. These double-stranded cDNA fragments were end-repaired by adding a single ‘A’ base and ligation of adapters. The adaptor modified fragments were selected by gel purification and amplified, through PCR, to create the final cDNA library. DGE sequencing was carried out on an Illumina HiSeq 2000 platform that generated 50 bp single-end raw reads.

### Analysis of DGE tags and bioinformatics by GO and KEGG

Raw reads generated by single-end sequencing were also submitted to the Genome Sequence Archive (GSA) database (http://gsa.big.ac.cn/index.jsp) with the BioProject identifier <PRJCA000207>. After trimming, the clean reads were mapped back onto the assembled transcriptome and the read count for each gene was derived from the mapping results obtained by RSEM, a user-friendly software package for quantifying gene and isoform abundances from single-end or paired-end RNA-Seq data. All read counts were normalized to reads per kilo bases per million mapped reads (RPKM). EdgeR was used to determine differential expressions^75^. Transcripts with an adjusted *p* value, 0.05 were accepted as being differential. Functional annotation and classification of genes were determined both by employing local protein blasts against Gene Ontology Consortium (http://geneontology.org/), Blast2GO (Bioinformatics Department, CIPF, Valencia, Spain), and Kyoto Encyclopedia of Genes and Genomes (KEGG) (http://www.genome.jp/kegg-bin/search_pathway).

### Protein extraction, digestion, iTRAQ labeling, HPLC fractionation and LC-MS/MS analysis

Two biological replicates of each sample were performed in the iTRAQ analysis. Proteins in either bile or intestinal mucus were extracted using the similar method for CSF samples^76^. Firstly proteins were precipitated by trichloroacetic acid, then measured using BCA assay, later proteins (200 μg) from each test sample were digested by trypsin (Promega, Madison, WI) at 37 °C for 12 h. The peptides were extracted using 50% ACN, 5% acetic acid. Tryptic peptides derived from each sample were combined separately and concentrated using a concentrator (Eppendorf AG, Hamburg, Germany). Then iTRAQ labeling was performed using iTRAQ Reagent Multi-Plex Kit (Applied Biosystem, Foster City, CA). The sample was then fractionated into fractions by high pH reverse-phase HPLC using Agilent 300Extend C18 column (5 μm particles, 4.6 mm ID, 250 mm length). Briefly, peptides were first separated with a gradient of 2% to 60% acetonitrile in 10 mM ammonium bicarbonate pH 10 over 80 min into 80 fractions, Then, the peptides were combined into 14 fractions and dried by vacuum centrifuging.

Then LC-MS/MS analysis was carried out. Peptides were dissolved in 0.1% FA, directly loaded onto a reversed-phase pre-column (Acclaim PepMap 100, Thermo Scientific). Peptide separation was performed using a reversed-phase analytical column (Acclaim PepMap RSLC, Thermo Scientific). The gradient was comprised of an increase from 6% to 23% solvent B (0.1% FA in 98% ACN) over 20 min, 23% to 35% in 12 min and climbing to 85% in 5 min then holding at 85% for the last 5 min, all at a constant flow rate of 300 nl/min on an EASY-nLC 1000 UPLC system. The resulting peptides were analyzed by Q ExactiveTM hybrid quadrupole-Orbitrap mass spectrometer (ThermoFisher Scientific). The peptides were subjected to NSI source followed by tandem mass spectrometry (MS/MS) in Q ExactiveTM (Thermo) coupled online to the UPLC. Intact peptides were detected in the Orbitrap at a resolution of 70,000. Peptides were selected for MS/MS using NCE setting as 30; ion fragments were detected in the Orbitrap at a resolution of 17,500. A data-dependent procedure that alternated between one MS scan followed by 20 MS/MS scans was applied for the top 20 precursor ions above a threshold ion count of 2E4 in the MS survey scan with 30.0 s dynamic exclusion. The electrospray voltage applied was 2.0 kV. Automatic gain control (AGC) was used to prevent overfilling of the ion trap; 5E4 ions were accumulated for generation of MS/MS spectra. For MS scans, the m/z scan range was 350 to 1600. Fixed first mass was set as 100 m/z. The resulting MS/MS data were processed using Mascot search engine (v.2.3.0). Tandem mass spectra were searched against uniprot_Oreochromis_niloticus.fasta database. Trypsin/P was specified as cleavage enzyme allowing up to 2 missing cleavages. Mass error was set to 10 ppm for precursor ions and 0.02 Da for fragment ions. Carbamidomethyl on Cys, iTRAQ-8 plex (N-term) and iTRAQ-8 plex (K) were specified as fixed modification and oxidation on Met was specified as variable modifications. FDR was adjusted to <1% and peptide ion score was set > 20.

### Protein identification, quantification and bioinformatics analysis by GO, KEGG and InterPro

Protein identification and quantification were performed following previously published protocol^77^. The relative protein ratios of different samples were calculated as the median of all peptides belonging to the assigned sample. Functional annotation and classification of all identified proteins were determined both by employing local protein blasts against Gene Ontology Consortium (http://geneontology.org/), Blast2GO (Bioinformatics Department, CIPF, Valencia, Spain), and Kyoto Encyclopedia of Genes and Genomes (KEGG) (http://www.genome.jp/kegg-bin/search_pathway). Identified proteins domain functional descriptions were annotated by InterProScan based on protein sequence alignment method, and the InterPro domain database was used. The mass spectrometry proteomics data have been deposited to the ProteomeXchange Consortium (http://proteomecentral.proteomexchange.org) via the MASSIVE with the dataset identifier <PXD003962>.

### Construction of tilapia immune gene library and following bioinformatics analysis

The tilapia immune gene library was set up based on tilapia gene information obtained via blasting each sequence to NCBI NR database (ftp://ftp.ncbi.nih.gov/blast/db/FASTA/nr.gz) (Fig. 1C). And the selection of tilapia immune genes was done by consulting many reviews on fish immunology, then the classification of tilapia immune genes followed the Shao’s approach^78^ with modifications. The tilapia immune gene library contains information for immune genes at two levels (Table 3). Nine categories of immune processes, including “acute phase reactions”, “pattern recognition genes”, “antigen processing and regulators”, “complement system”, “inflammatory cytokines and receptors”, “adapters, effectors and signal transducers”, “innate immune cells related”, “T cell and B cell antigen activation”, as well as “other genes related to immune cell response”, were used at the first level, and then many categories of immune genes for each immune process were described at the second level (Table S4). Afterwards (Fig. 1C), the library was applied to filter either transcriptomic or proteomic results in order to obtain detailed information of immune process as well as particular immune genes in fish gut and liver, and then Venn analysis was also done to explore key immune transcripts or proteins.

### Real-time quantitative reverse-transcription PCR verification

Real-time qPCR was performed in a DNA Engine Chromo 4 real-time system (BioRad) with SYBR green real-time PCR master mix (BioRad). The expression of genes was calculated as relative expression to β-actin using the 2(-ΔΔC(T)) method and samples were analyzed in triplicates^43^. Briefly, first the β-actin transcript maintained stable under the treatments analyzed by semi-quantitative PCR (data were shown), then the reactions were performed in a 10 ul mixture containing 5 ul SsoAdvanced™ Universal SYBR^®^ Green Supermix (Biorad), 250 nM primers, and 1 ul cDNA, and the thermocycling was conducted as follows: 95 °C for 2 min, then 45 temperature cycles (95 °C 30 s; 60 °C, 60 s), later the relative expression levels (fold change) of the tested genes, were calculated using the relative expression software tool (Biorad). The primers used for qPCR were listed in Table S2.

### Statistics and correlation analysis

T-test was used to assess differences, with FDR adjusted *p* < 0.05 for DGE data meanwhile *p* < 0.01 for iTRAQ data. Qualitative comparisons were made between different tissues or fluids by counting No. of genes differentially expressed. For comparisons between same tissues or fluids, the differential expressed genes were analyzed according to the fold change (for DGE) or ratio (for iTRAQ). Then the data were rearranged in EXCEL and were applied to plot charts using R scripts.

## Additional Information

**How to cite this article**: Wu, N. *et al*. Fish gut-liver immunity during homeostasis or inflammation revealed by integrative transcriptome and proteome studies. *Sci. Rep.* **6**, 36048; doi: 10.1038/srep36048 (2016).

**Publisher’s note**: Springer Nature remains neutral with regard to jurisdictional claims in published maps and institutional affiliations.

## Supplementary Material

Supplementary Dataset 1

Supplementary Dataset 2

Supplementary Dataset 3

Supplementary Dataset 4

Supplementary Dataset 5

Supplementary Dataset 6

Supplementary Dataset 7

Supplementary Dataset 8

Supplementary Dataset 9

Supplementary Dataset 10

Supplementary Dataset 11

## Figures and Tables

**Table 1:** Immune related GO terms and KEGG pathways.

	GO TERMS			KEGG PATHWAYS
	Biological process	Cellular component	Molecular function	
Gut advantage	Integrin-mediated signaling pathway (0 h); Antigen processing and presentation via MHC class II (0 h, 12 h, 36 h); Antigen processing and presentation (0 h, 12 h, 36 h); Immune response (0 h, 12 h, 36 h); Immune system process (0 h,12 h, 36 h);	Integrin complex (0 h); MHC class II protein complex (0 h, 12 h, 36 h); MHC protein complex (0 h, 12 h, 36 h);	Cytokine activity (12 h);	Cell adhesion molecules (0 h, 12 h, 36 h); Intestinal immune network for IgA production (0 h, 12 h); Phagosome (0 h, 12 h, 36 h);
Liver advantage	Integrin-mediated signaling pathway (0 h); Immune response (0 h, 12 h, 36 h); Immune system process (0 h, 12 h, 36 h);	Integrin complex (0 h);	Cytokine activity (12 h);	Cell adhesion molecules (0 h, 12 h, 36 h); Phagosome (0 h, 12 h, 36 h);
Mucus advantage	Immune system process (0 h);	—	—	Antigen processing and presentation (36 h); Complement and coagulation cascades (36 h);
Bile advantage	Immune system process (0 h, 36 h);	—	—	Complement and coagulation cascades (0 h);

**Table 2:** Immune related predicted protein domains.

Predicted protein domains	
Mucus advantage (23)	Immunoglobulin I-set (36 h); Immunoglobulin E-set (0 h); Apolipoprotein A1/A4/E (36 h); Immunoglobulin-like domain (36 h); α-2-macroglobulin, conserved site (36 h); α-macroglubulin, receptor-binding (36 h); Complement C1r-like EGF domain (36 h); α-2-macroglobulin, thiolester bond-forming (36 h); Immunoglobulin subtype (36 h); EGF-like,conserved site (36 h); Membrane attack complex component/perforin/complement C9 (36 h); Immunoglobulin-like fold (36 h); Membrane attack complex component/perforin domain, conserved site (36 h); EGF-like calcium-binding domain (36 h); EGF-like calcium-binding, conserved site (36 h); EGF-type aspartate/asparagines hydroxylation site (36 h); C-type lectin fold (36 h); Coagulation factor, subgroup, Gla domain (36 h); C-type lectin fold (36 h); C-type lectin (36 h); Leucine-rich repeat-containing N-terminal (36 h); C-type lectin-like (36 h)
Bile advantage (30)	Immunoglobulin I-set (0 h); Fibringen,α/β/γ chain, C-terminal globular,subdomain 1 (0 h, 36 h); Fibringen,α/β/γ chain, C-terminal globular,subdomain 2 (0 h, 36 h); Fibrinogen,α/β/γ chain, C-terminal globular domain (0 h, 36 h); Fibrinogen, α/β/γ chain, coiled coil domain (0 h, 36 h); Fibrinogen, conserved site (0 h, 36 h); Apolipoprotein A1/A4/E (0 h); Immunoglobulin-like domain (0 h); α-2-macroglobulin, conserved site (0 h); α-macroglubulin, receptor-binding (0 h); Complement C1r-like EGF domain (0 h); α-2-macroglobulin,thiolester bond-forming (0 h); α-2-macroglubulin, N-terminal 2 (0 h); α-macroglobulin complement component (0 h); α-2-macroglobulin (0 h); α-2-macroglobulin,N-terminal (0 h); Immunoglobulin subtype (0 h); EGF-like,conserved site (0 h); Membrane attack complex component/perforin/complement C9 (0 h); Immunoglobulin-like fold (0 h); Membrane attack complex component/perforin domain, conserved site (0 h); EGF-like calcium-binding domain (0 h); EGF-like calcium-binding, conserved site (0 h); EGF-type aspartate/asparagines hydroxylation site (0 h); C-type lectin fold (0 h); Coagulation factor, subgroup, Gla domain (0 h); C-type lectin fold (0 h); C-type lectin (0 h); Leucine-rich repeat-containing N-terminal (0 h); C-type lectin-like (0 h);

**Table 3:** Description of the tilapia immune gene library.

Immune process	Total	Annotated	Blasted Homologue
Acute phase reactions	47	27	20
Pattern recognition genes	549	345	204
Antigen processing and regulators	192	88	104
Complement system	78	36	42
Inflammatory cytokines and receptors	532	422	110
Adapters, effectors and signal transducers	286	217	69
Innate immune cells related	42	34	8
T cell and B cell antigen activation	430	273	157
Other genes related to immune cell response	605	400	205
	2761	1842	919

Notes: the numbers of total genes, annotated ones, as well as blasted homologue, are listed for each immune process.

**Table 4:**
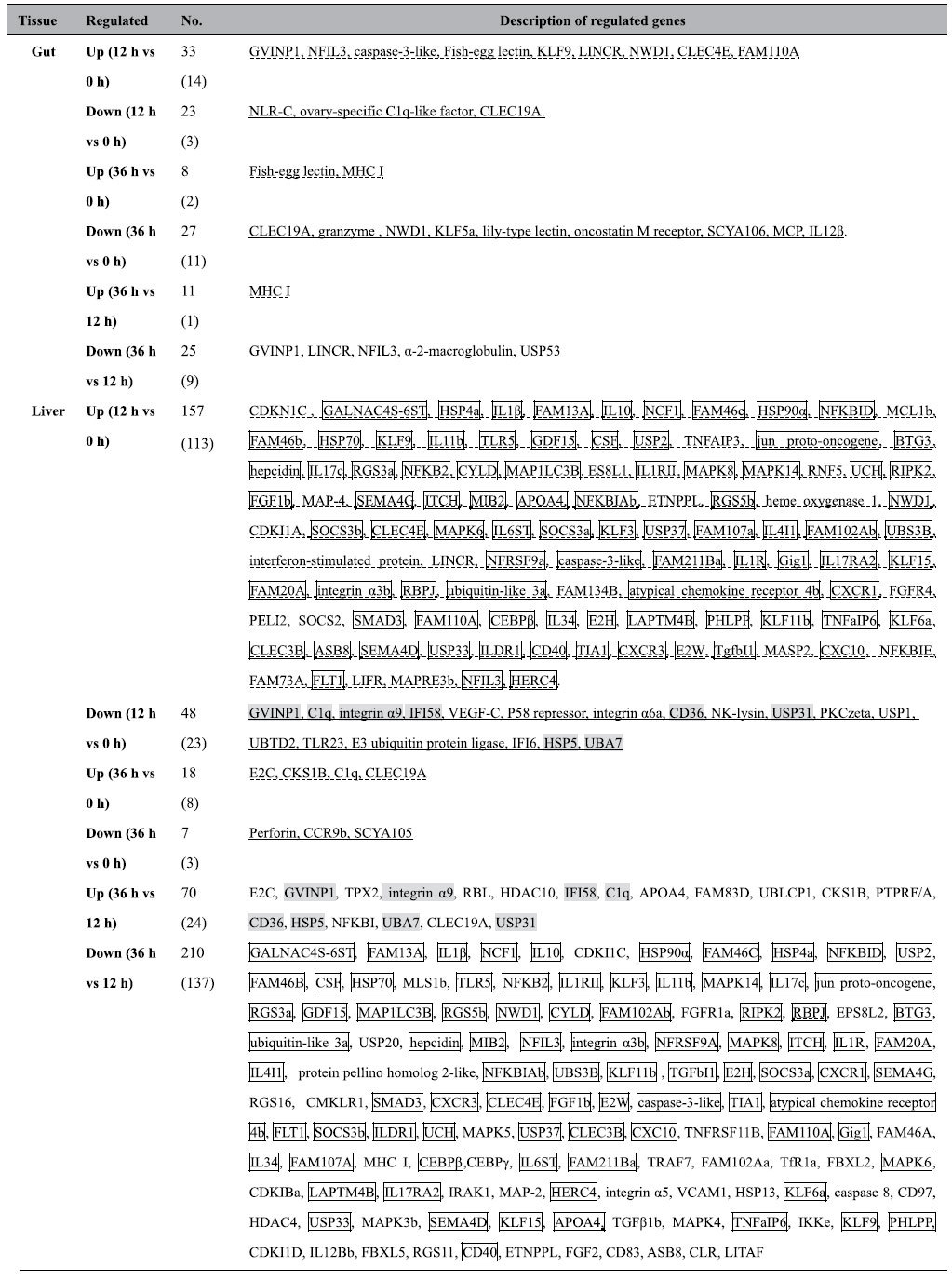
Regulated immune transcripts in gut or liver comparing steady and inflammational states.

Notes: the underlined immune transcripts may be related to homeostasis, whereas the dash-lined ones may be related to inflammation. The overlapped 84 transcripts in liver between up-regulated (12 h vs 0 h) and down-regulated (36 h vs 12 h) were in box, and the overlapped 8 ones between down-regulated (12 h vs 0 h) and up-regulated (36 h vs 12 h) were in shade box. The most differentiated transcripts (adjusted *P* value < 0.01, highlighted in Table S10), with their No. in the brackets following the total No., were listed in the sequence following the inverted order of log2 fold change.

**Table 5:**
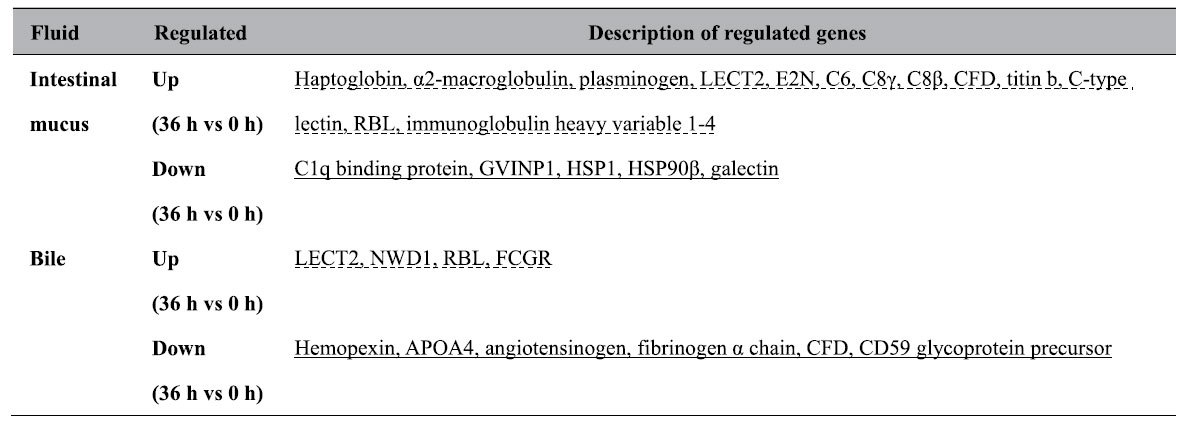
Regulated proteins in intestinal mucus or bile comparing steady and inflammational states.

Notes: the underlined immune proteins may be related to homeostasis, whereas the dash-lined ones may be related to inflammation. The most differentiated proteins (*P* value < 0.01, all in Table S11) were listed, with the sequence following the inverted order of ratio.
