# Supplementary material for: Fish gut-liver immunity during homeostasis or inflammation revealed by integrative transcriptome and proteome studies
Source: Sci Rep. 2016 Nov 3;6:36048. doi: 10.1038/srep36048 (PMC5093735; doi:10.1038/srep36048)
Supplement: Supplementary Dataset 1 [file srep36048-s1.doc]

Figure S1

A


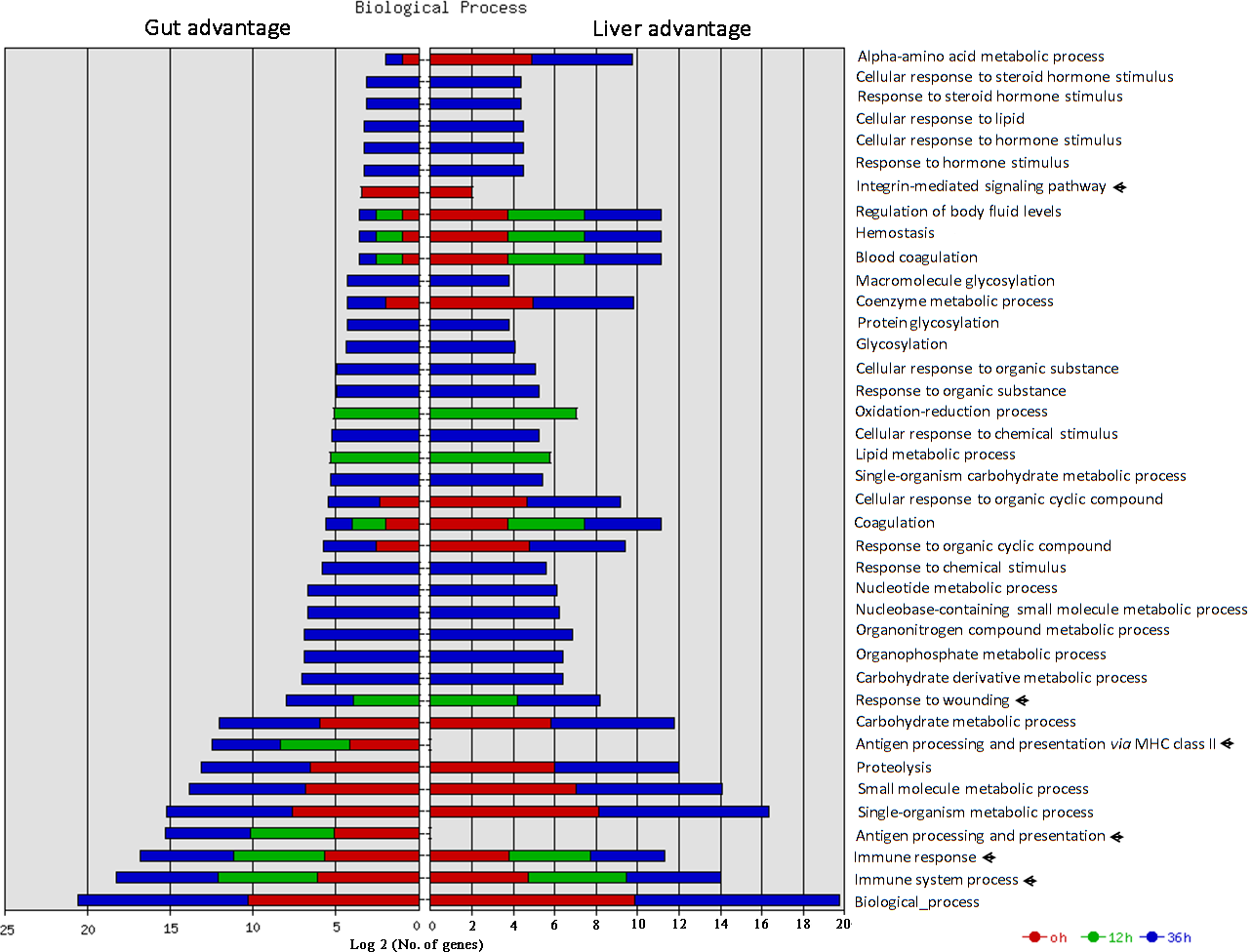


B


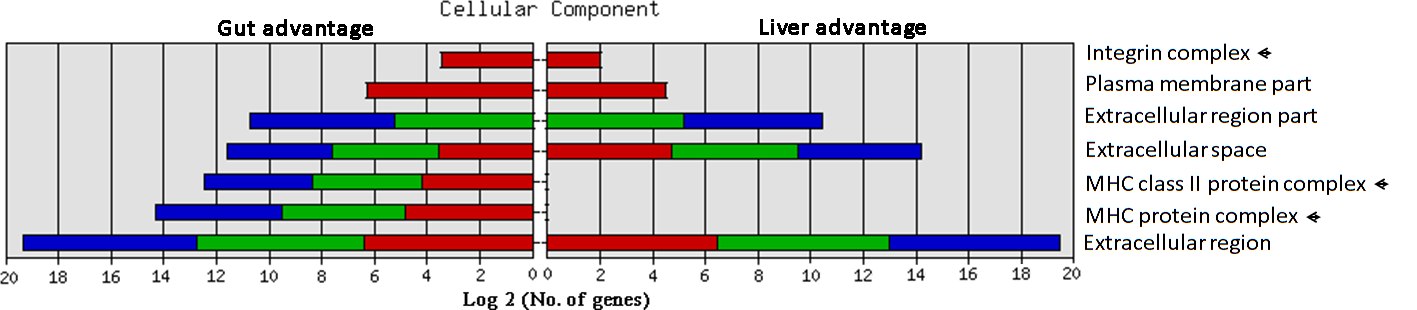


**Fig. S1 Involved GO terms and KEGG pathways of differential expressed transcripts between gut and liver.** (A) Go terms in biological process; (B) Go terms in cellular component; (C) Go terms in cellular component; (D) KEGG pathways. The immune related terms were labeled with arrows. The bars were labeled in red, green, and blue, for 0 h, 12 h, and 36 h, respectively.

Figure S1

C


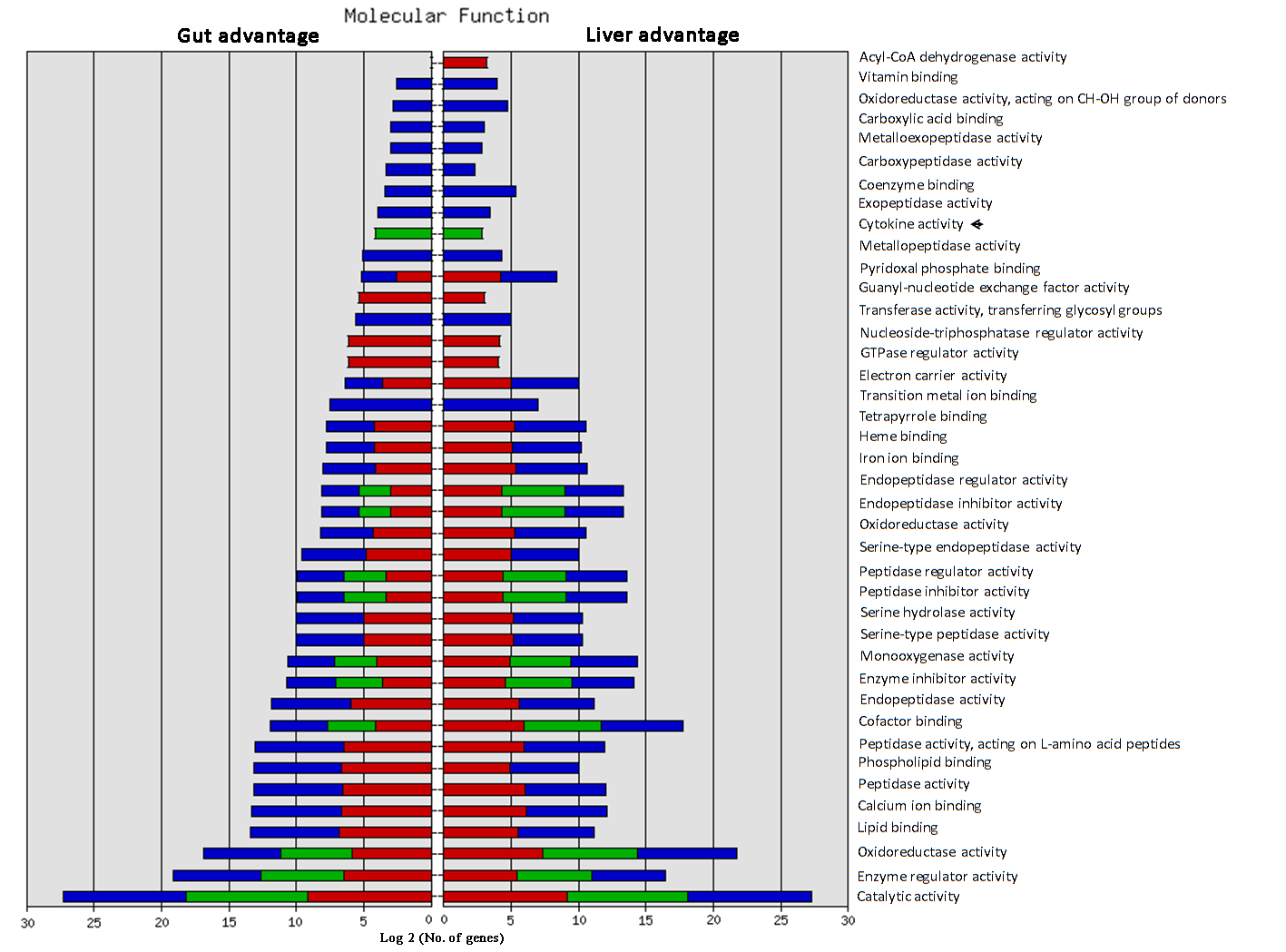


D


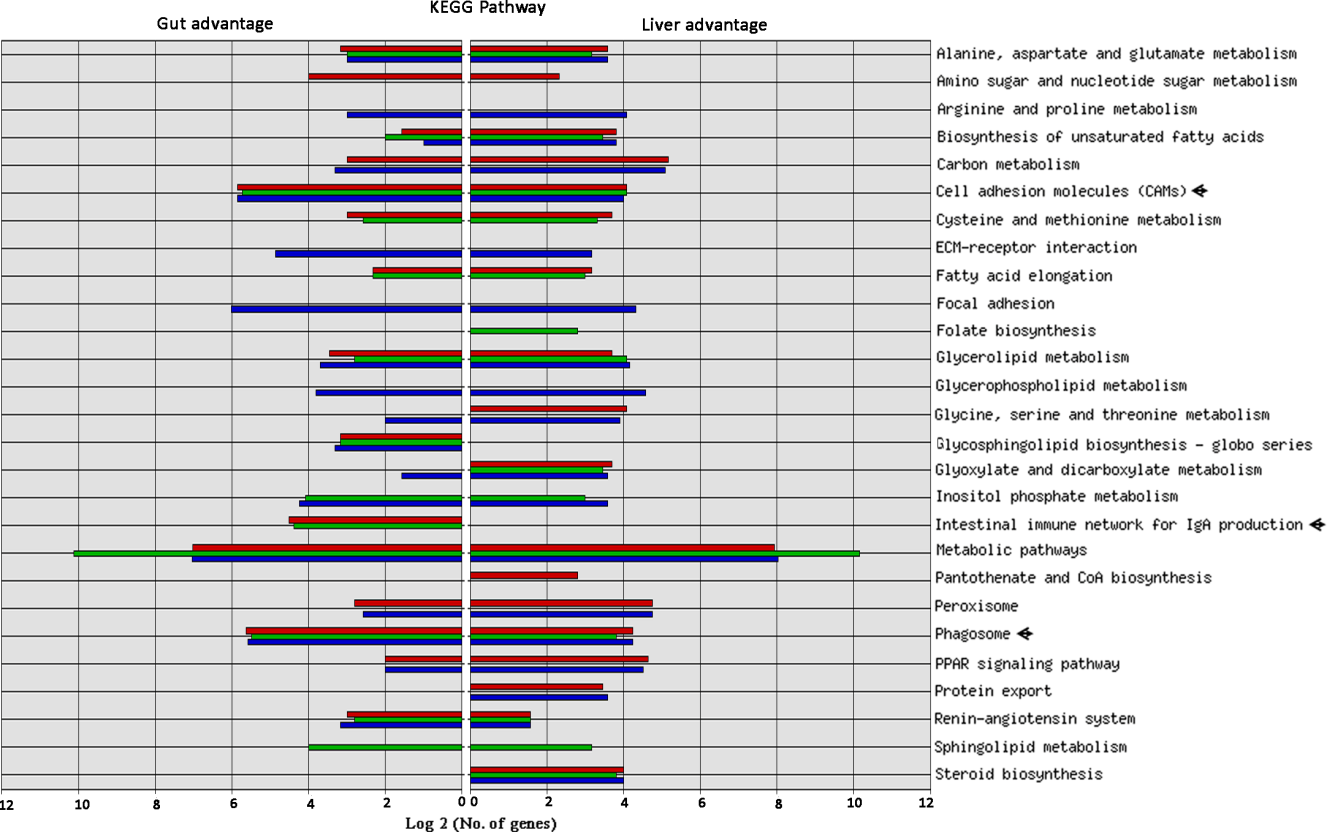


Figure S2

A


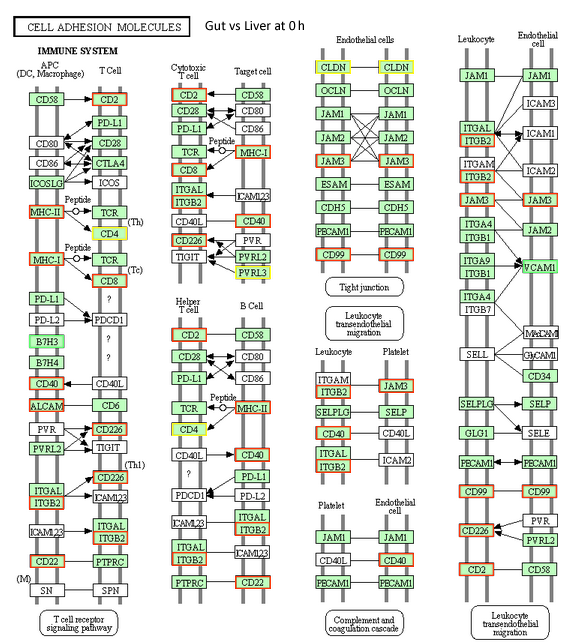


Figure S2

B


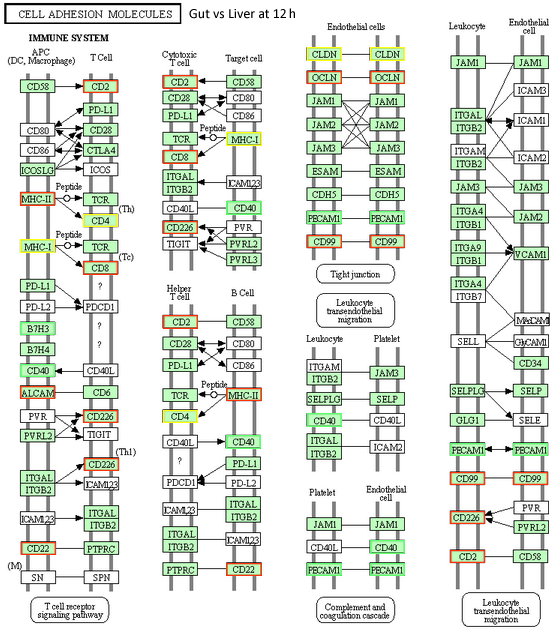


Figure S2

C


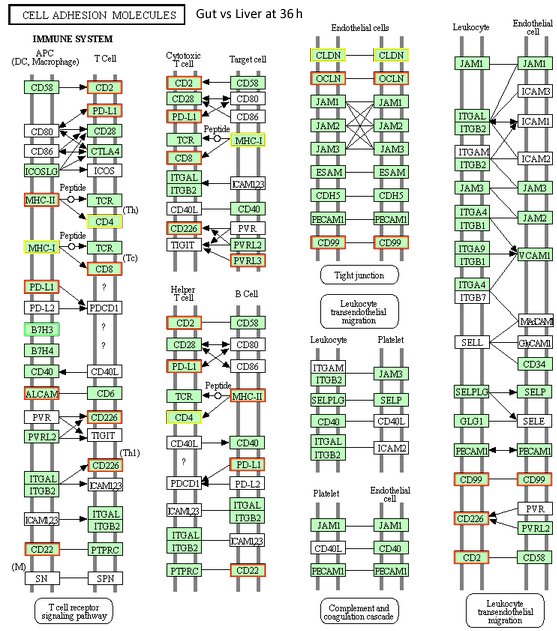


Figure S2

D


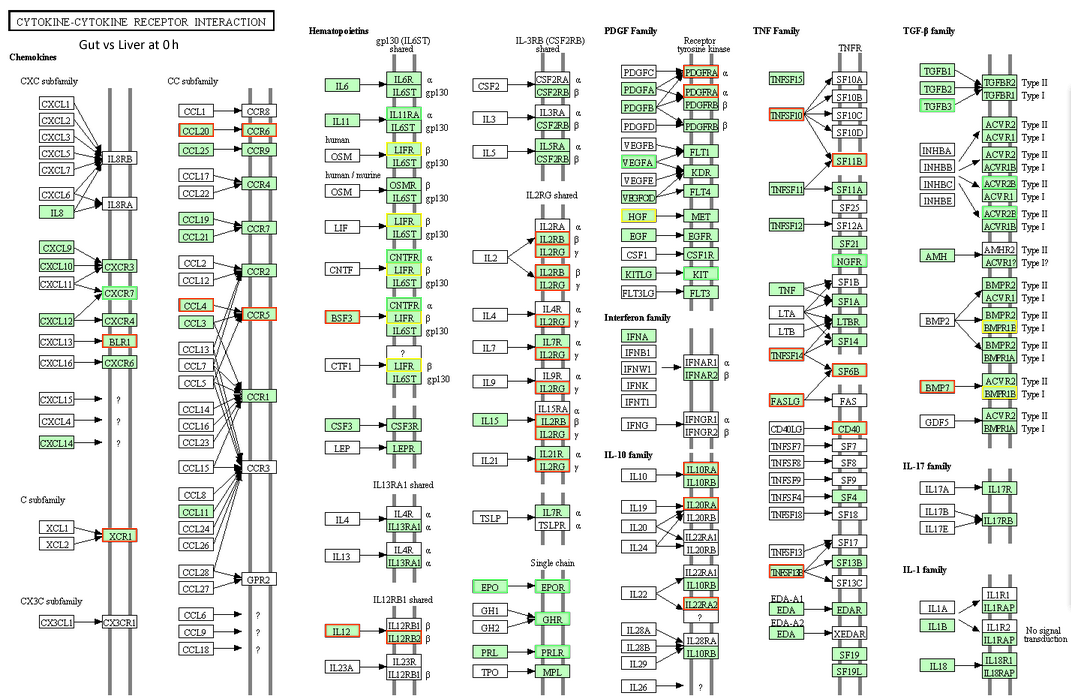


Figure S2

E


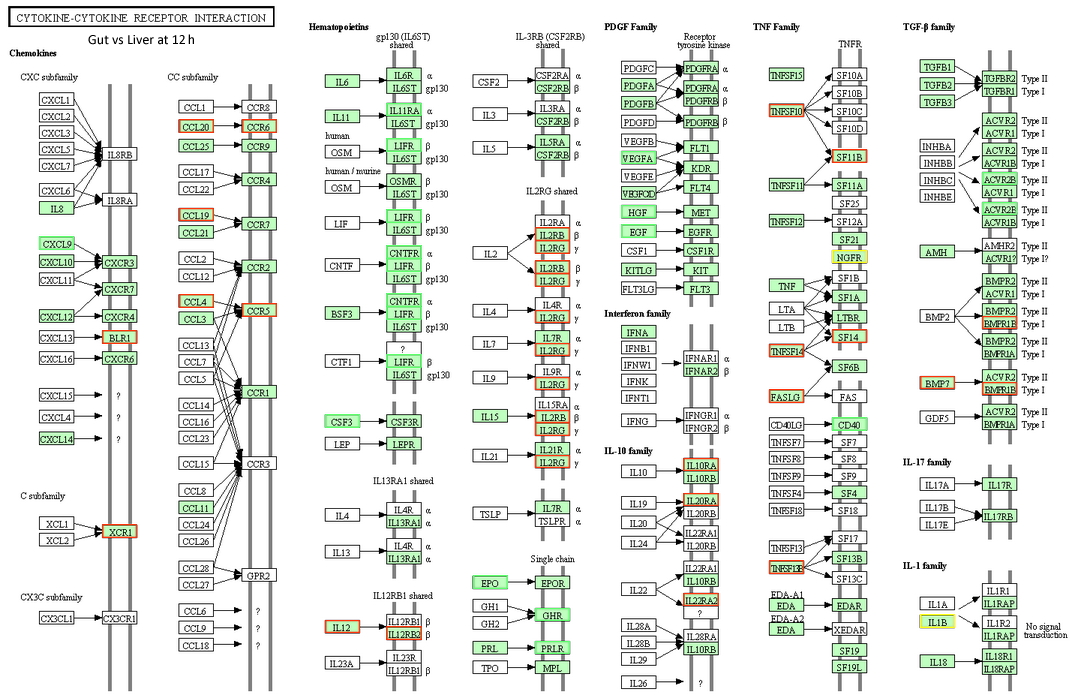


Figure S2

F


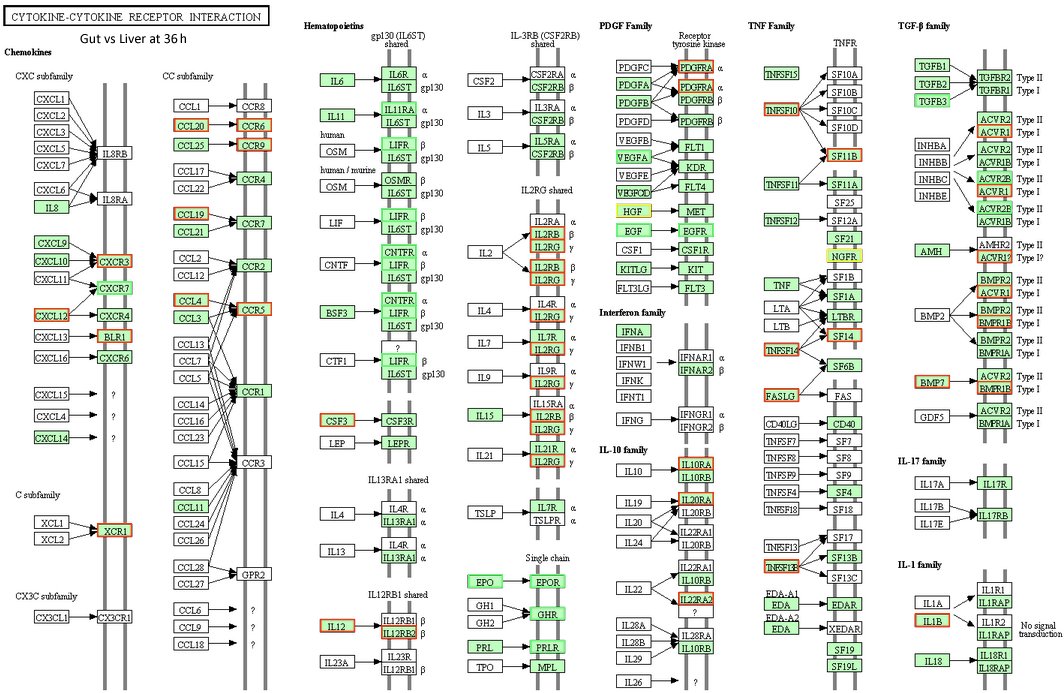


Figure S2

G


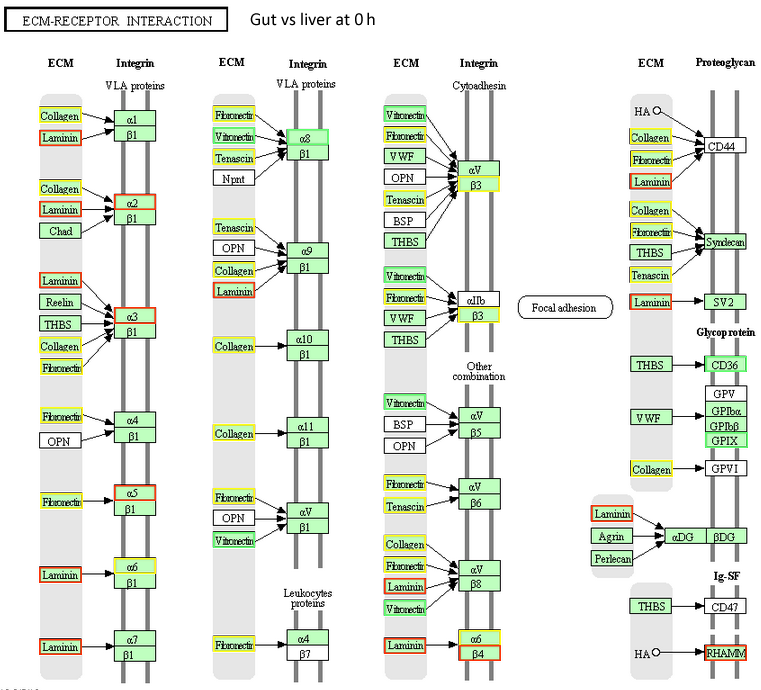


Figure S2

H


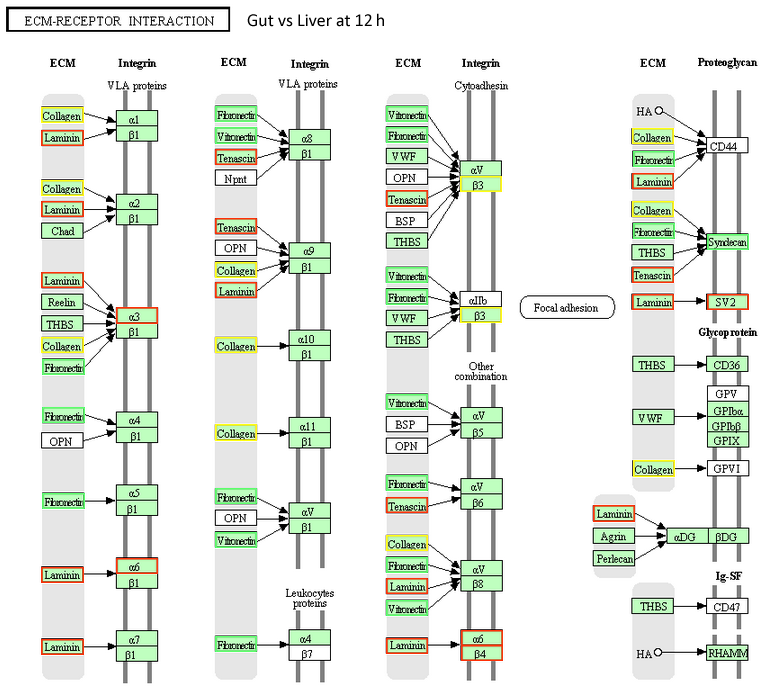


Figure S2

I


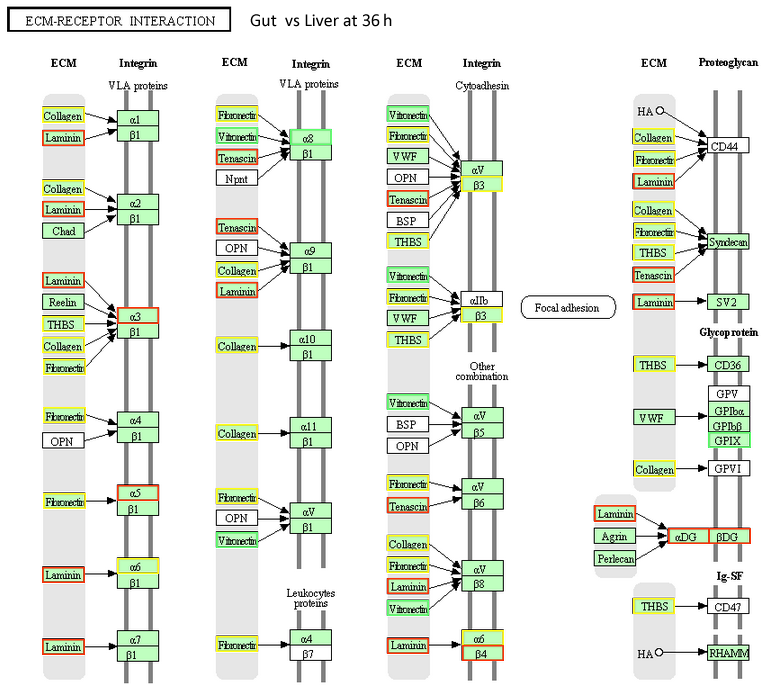


**Fig. S2 Mostly matched immune related part of KEGG pathways by comparing gut and liver DGE data at different time points of either healthy or inflammational stages.** (A-C, A: 0 h; B: 12 h; C: 36 h) In **t**he matched pathway “cell adhesion molecules”, the immune related cell adhesion interfaces include APC & T cell, cytotoxic T cell & target cell, helper T cell & B cell, endothelial cells, leukocyte & platelet, platelet & endothelial cell, and leukocyte & endothelial cell. And, interestingly, the leukocyte transendothelial migration was revealed very highly gut advantage only at 0h. (D-F) In the matched pathway “cytokine-cytokine receptor interaction” (D: 0 h; E: 12 h; F: 36 h), the chemokines were found mostly significantly regulated among 3 time points. (G-I) In the matched pathway “ECM-receptor interaction” (G: 0 h; H: 12 h; I: 36 h), the ECM-integrin interactions were found regulated significantly in fish gut-liver immunity among 3 time points. The boxes filled by green indicated the genes could be recognized. The boxes with red frame indicate gut-advantage genes, and ones with green frame indicate liver-advantage genes, while ones with yellow frame indicate transcripts containing both gut- and liver-advantage among 3 replicates.

Figure S3

A


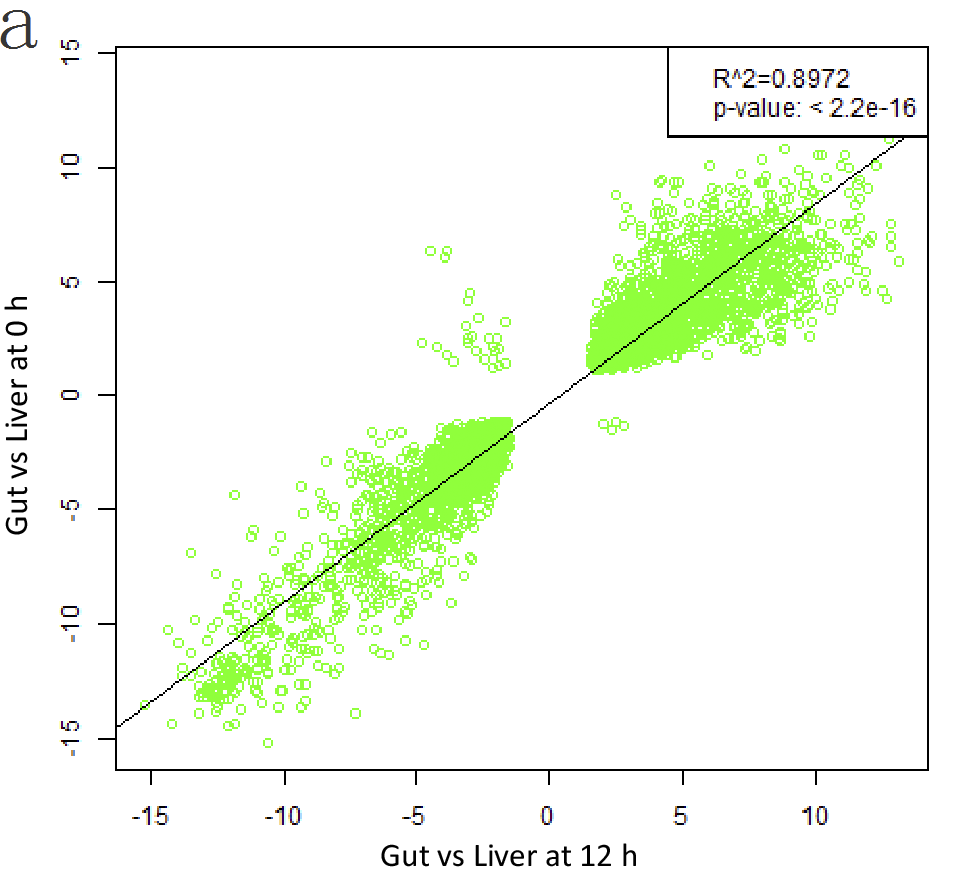

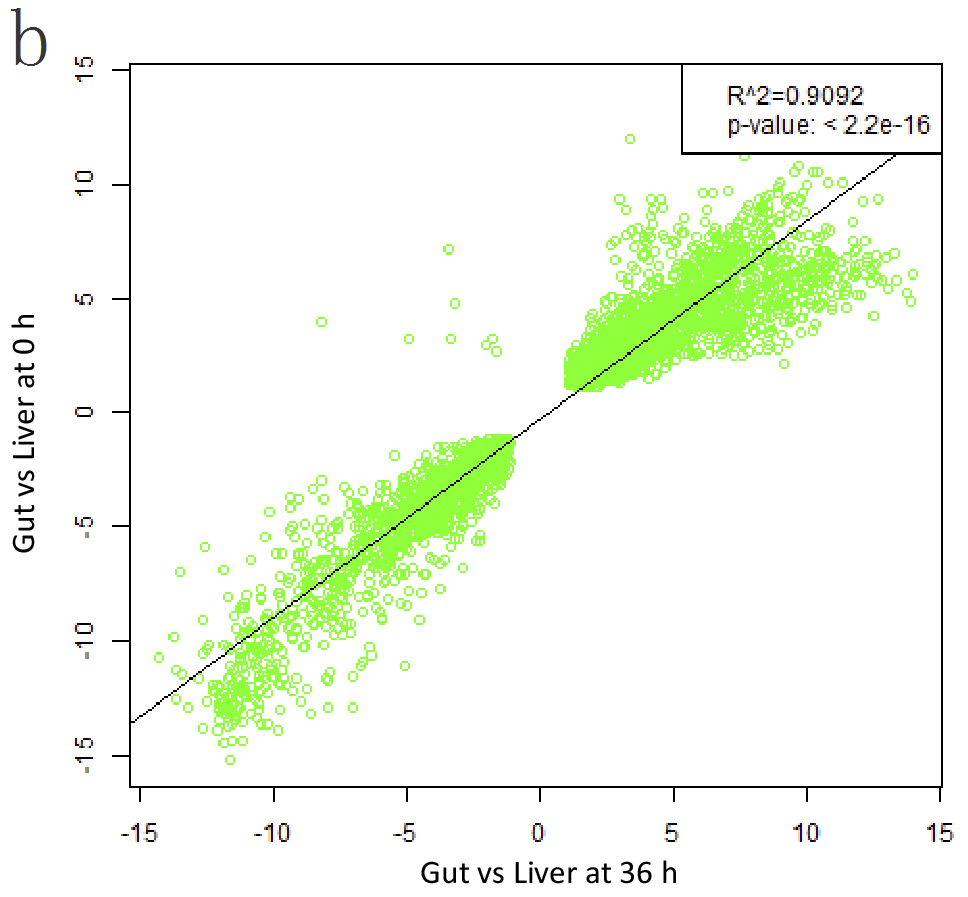


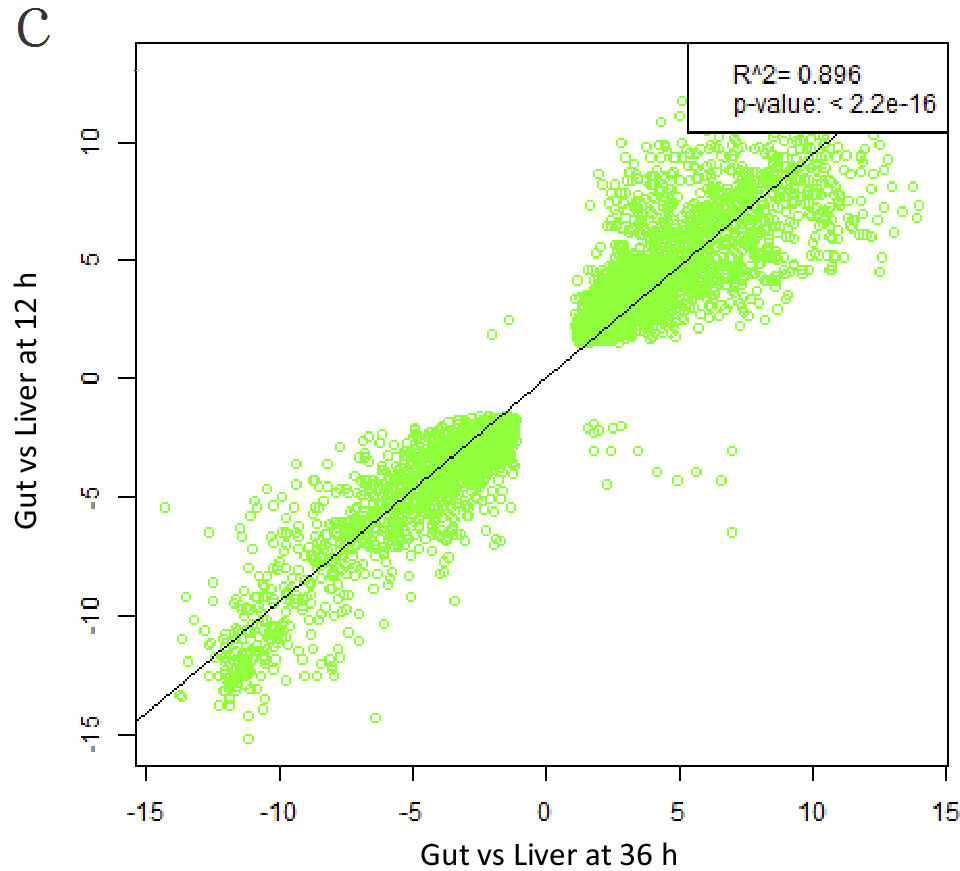

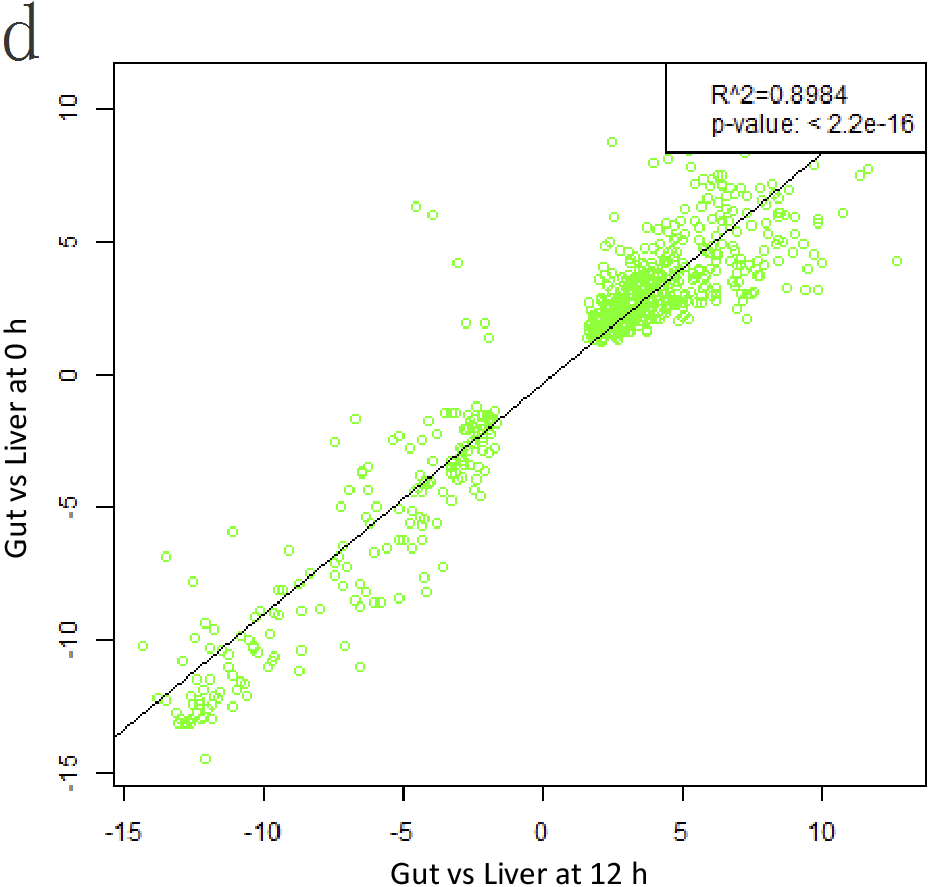


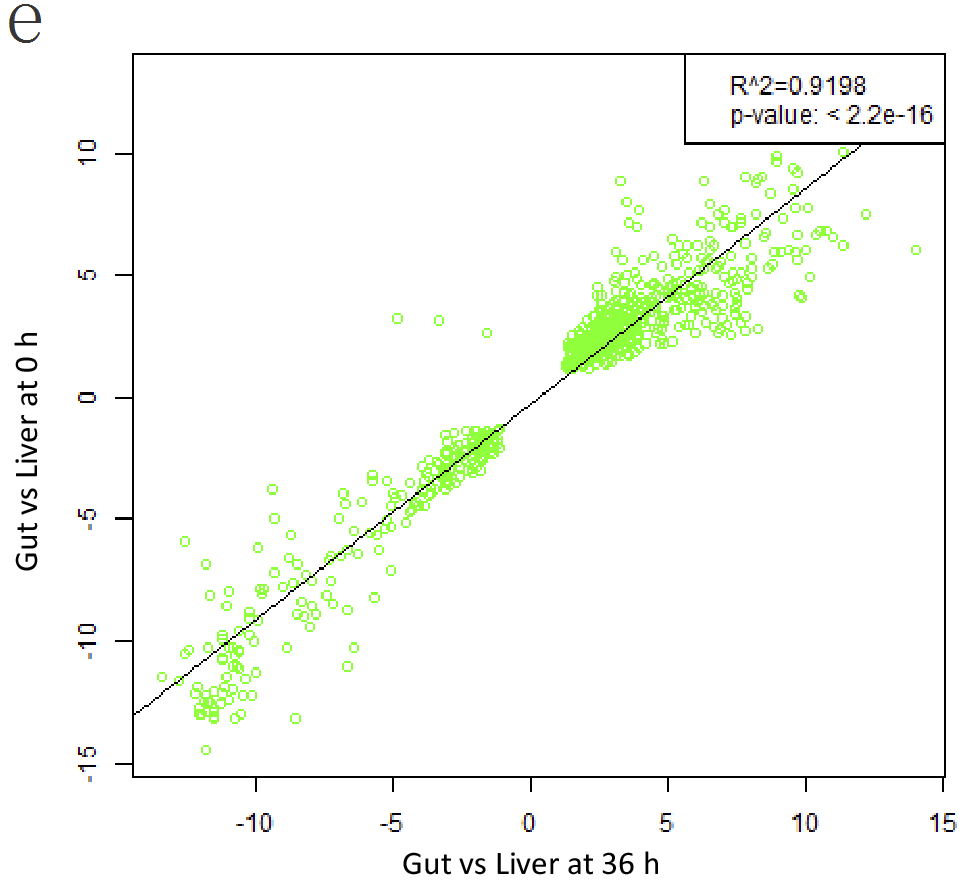

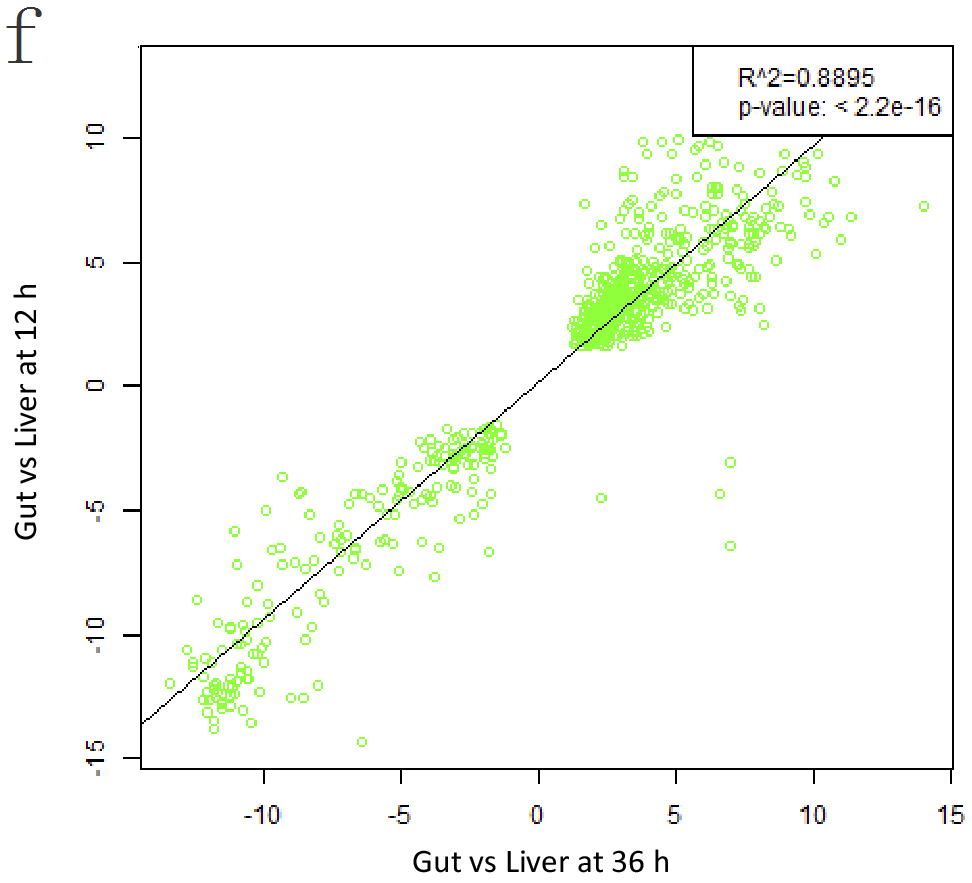


Figure S3

B


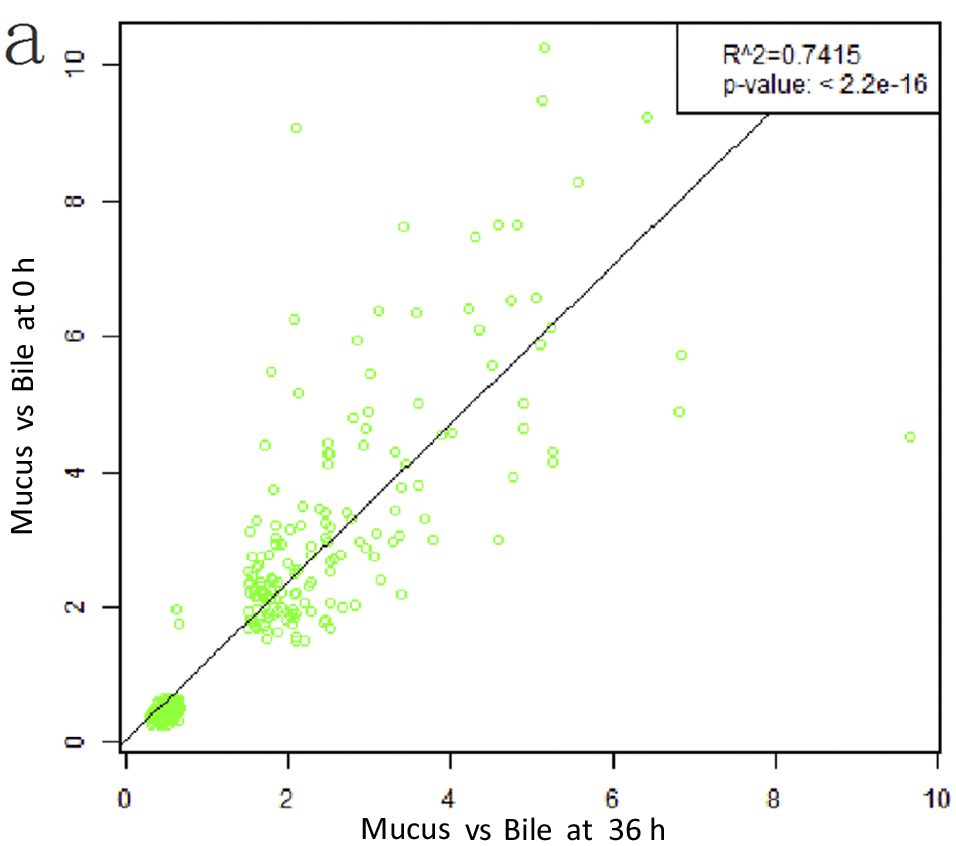

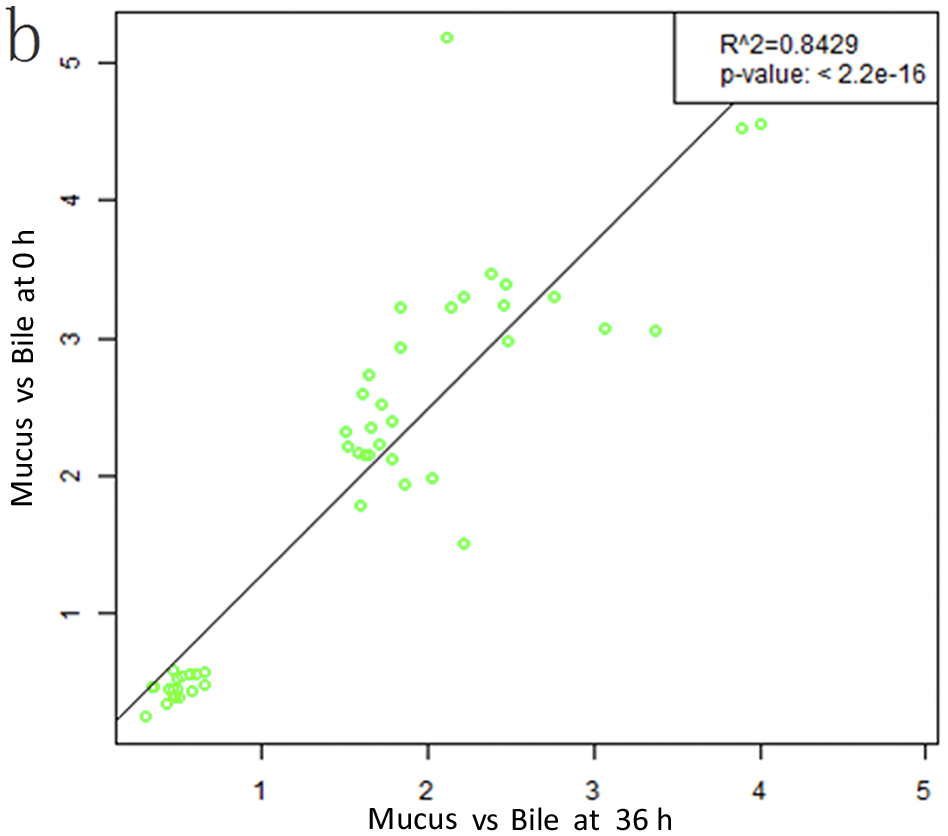


**Fig. S3** [**Correlation**](javascript:void(0);) [**analysis**](javascript:void(0);) **among DGE or iTRAQ data of different time point.** (A) [Correlation](javascript:void(0);) among DGE data of 0 h, 12 h and 36 h, for differential genes between gut and liver, a-c: all differential genes; d-f: differential immune genes. (B) [Correlation](javascript:void(0);) of iTRAQ data of 0 h and 36 h, for differential protein between bile and intestinal mucus: a) all differential protein; b) differential immune protein.

Figure S4


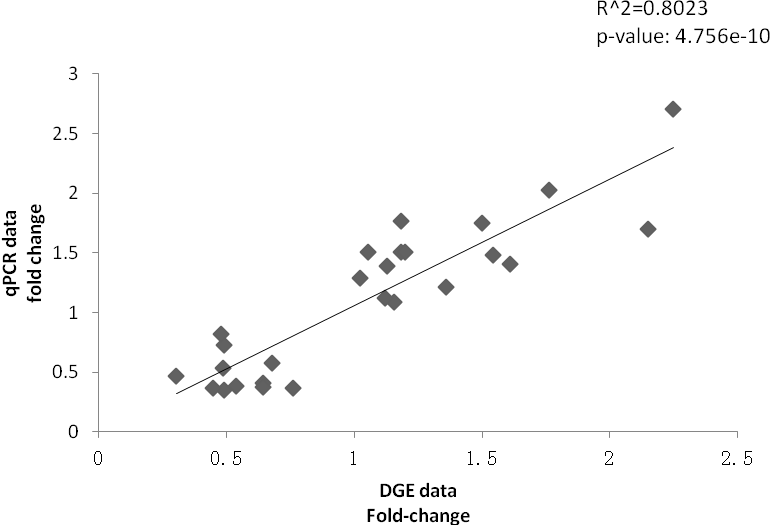


**Fig. S4 Correlation analysis between fold changes of both qPCR and DGE data for validation.** Among 26 reactions of 14 genes (detailed in Table S2), fold-changes between DGE and qPCR results correlated well (R^2=0.8023).

Figure S5

A


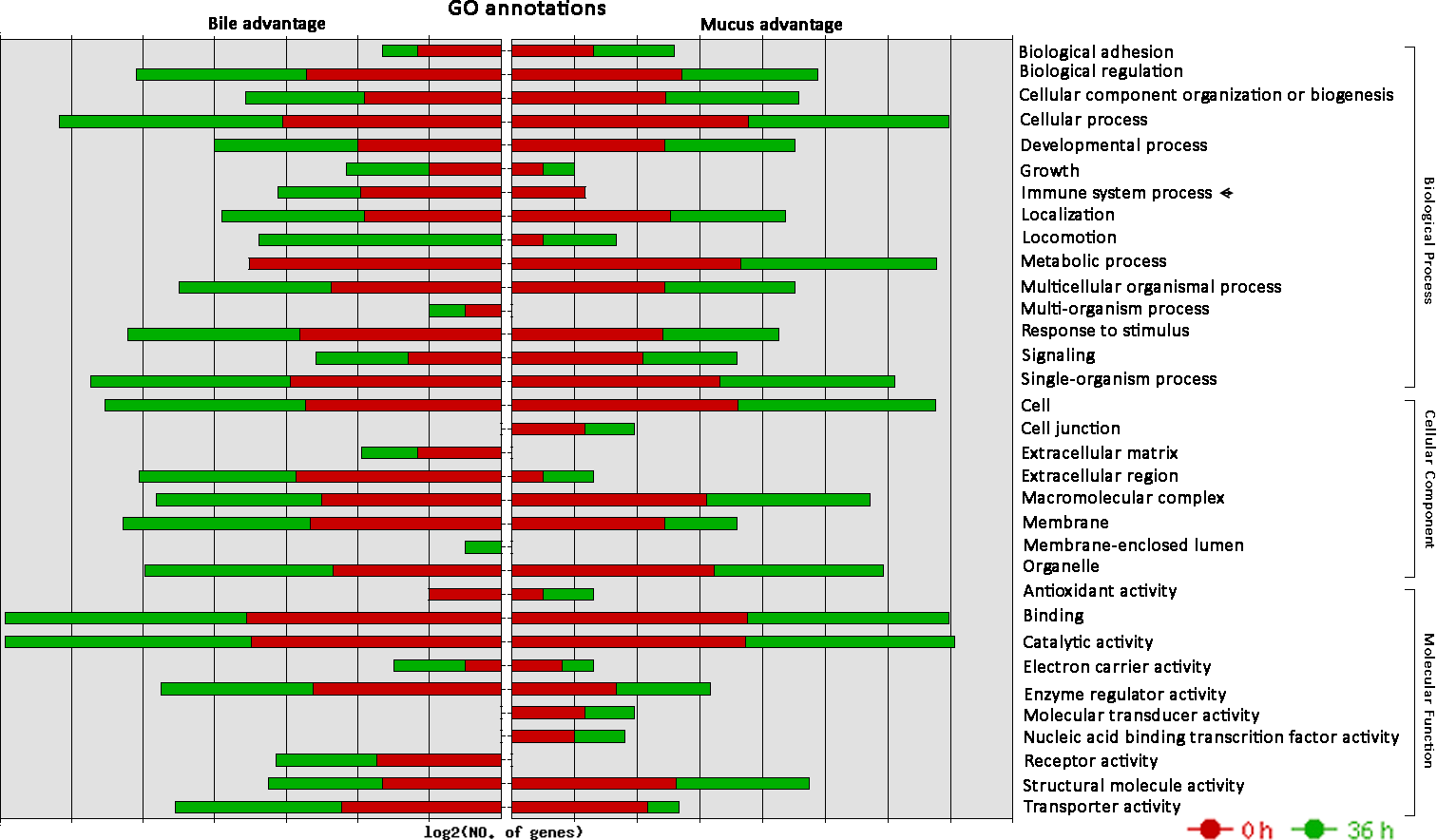


B


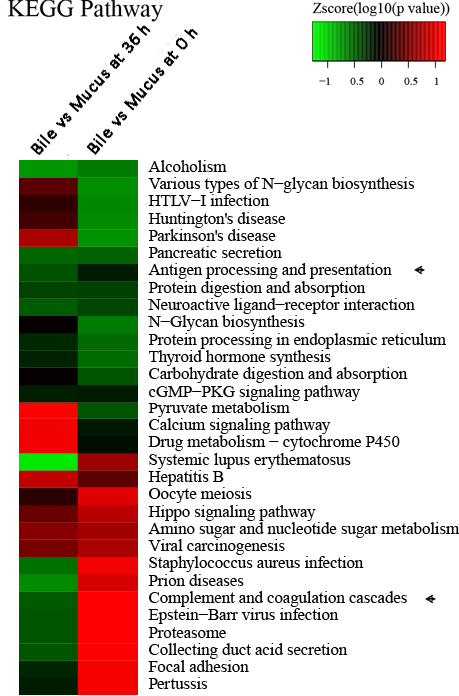


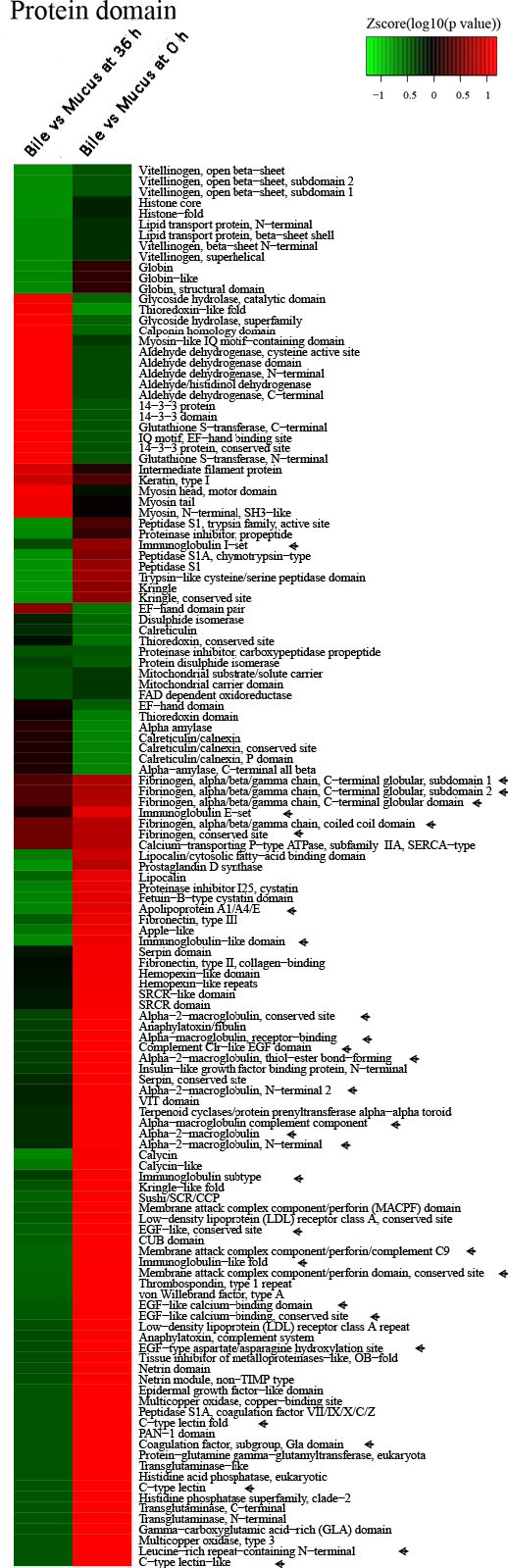


C

**Fig. S5 Involved GO terms, KEGG pathways, and predicted protein domains of differential proteins between intestinal mucus and bile.** (A) Involved GO terms, including those in biological process, cellular component, and cellular component. The bars were labeled in red and green for 0 h and 36 h respectively. (B) Hot map of involved KEGG pathways. (C) Hot map of predicted protein domains. The immune related terms, pathways, or domains, were labeled with arrows.

Table S1 Detailed information for quality of DGE data

| **Sample** | **No. of raw reads** | **No. of clean reads** | **No. of**  **total mapped reads (%)** | **No. of**  **multiple mapped (%)** | **No. of**  **unique mapped (%)** |
| --- | --- | --- | --- | --- | --- |
| Gut (0 h) -1 | 13400490 | 13375509 | 10841954 (81.06%) | 564198 (4.22%) | 10277756 (76.84%) |
| Gut (0 h) -2 | 12640224 | 12615352 | 10150090 (80.46%) | 548709 (4.35%) | 9601381 (76.11%) |
| Gut (0 h) -3 | 12966454 | 12942781 | 10597378 (81.88%) | 657807 (5.08%) | 9939571 (76.8%) |
| Gut (12 h) -1 | 12069850 | 12040680 | 9649046 (80.14%) | 584795 (4.86%) | 9064251 (75.28%) |
| Gut (12 h) -2 | 11301136 | 11278813 | 9209411 (81.65%) | 518169 (4.59%) | 8691242 (77.06%) |
| Gut (12 h) -3 | 14619618 | 14589186 | 12120068 (83.08%) | 590133 (4.05%) | 11529935 (79.03%) |
| Gut (36 h) -1 | 12741192 | 12704064 | 10277713 (80.9%) | 484528 (3.81%) | 9793185 (77.09%) |
| Gut (36 h) -2 | 12137306 | 12100511 | 9806105 (81.04%) | 490934 (4.06%) | 9315171 (76.98%) |
| Gut (36 h) -3 | 12076235 | 12052693 | 9669891 (80.23%) | 651100 (5.4%) | 9018791 (74.83%) |
| Liver (0 h) -1 | 11937965 | 11915576 | 9667463 (81.13%) | 491434 (4.12%) | 9176029 (77.01%) |
| Liver (0 h) -2 | 11091270 | 11062708 | 9108476 (82.33%) | 587723 (5.31%) | 8520753 (77.02%) |
| Liver (0h) -3 | 12090430 | 12058384 | 9829304 (81.51%) | 530030 (4.4%) | 9299274 (77.12%) |
| Liver (12 h) -1 | 12050056 | 12023482 | 9983263 (83.03%) | 442158 (3.68%) | 9541105 (79.35%) |
| Liver (12 h) -2 | 11415292 | 11391698 | 9403728 (82.55%) | 380609 (3.34%) | 9023119 (79.21%) |
| Liver (12 h) -3 | 12621163 | 12597399 | 10580286 (83.99%) | 444802 (3.53%) | 10135484 (80.46%) |
| Liver (36 h) -1 | 12363503 | 12308791 | 10214511 (82.99%) | 519493 (4.22%) | 9695018 (78.76%) |
| Liver (36 h) -2 | 11095037 | 11073091 | 8792822 (79.41%) | 499635 (4.51%) | 8293187 (74.89%) |
| Liver (36 h) -3 | 11091110 | 11061316 | 8914556 (80.59%) | 517701 (4.68%) | 8396855 (75.91%) |

Table S2 Detail information for qPCR validation

| **Gene ID & Description** | **Primers** | **Compared** | **FC in qPCR** | **FC in DGE** |
| --- | --- | --- | --- | --- |
| ENSONIG00000019808  (CD59) | F: 5'GACAGTCCTTTCAATCACGATGA 3';  R: 5'CGCAAACATTCAGTGTAGGTCTT 3' | Liver (12 h / 0 h) | 1.76 | 1.18 |
|  |  | Liver (36 h / 0 h) | 1.29 | 1.02 |
| ENSONIG00000014025  (CCR9) | F: 5'ATCGTCGCCACACTGCTCAAG 3' ; R: 5'ACCAGCACGCTGTTGTAAGGC 3' | Gut (12 h / 0 h) | 0.57 | 0.68 |
| ENSONIG00000011123  (C9) | F: 5'CCATACATACGACGGACAGTTTA 3'  R: 5'CTCTCATACTAACAGCACTTTGTA 3' | Liver (12 h / 0 h) | 1.09 | 1.15 |
|  |  | Liver (36 h / 0 h) | 2.02 | 1.76 |
| ENSONIG00000015284  (TGFβ) | F: 5'AACAGAATCTCCCTCATCTCATC 3';  R: 5'ATCCACTTCCAGTCCAAATCAC 3' | Liver (36 h / 0 h) | 1.70 | 2.15 |
|  |  | Liver (12 h / 0 h) | 0.54 | 0.49 |
| ENSONIG00000016817  (STAT4) | F: 5'TTGATTTCCGCTACAAGACCCT 3';  R: 5'GTCAAGCCGATTTAGCATTTCCT 3' | Liver (12 h / 0 h) | 1.51 | 1.18 |
|  |  | Liver (36 h / 0 h) | 0.39 | 0.54 |
| ENSONIG00000013024 (CD4) | F: 5'CATCGCCCATCCAAGACCAGA 3';  R: 5'ACGATGTTTTAGGTGAAGTCCAA 3' | Gut (12 h / 0 h) | 1.12 | 1.12 |
|  |  | Gut (36 h / 0 h) | 0.41 | 0.64 |
| ENSONIG00000011230  (IL1β) | F: 5'TGGTCTGATTGTCGTCCTGTCT 3';  R: 5'AAATGTCATCATGGTATTGCTCC 3' | liver (36 h / 12 h) | 1.22 | 1.36 |
| ENSONIG00000006056  (IL10R) | F: 5'GCTCTGCTTTGTGCTCTCCTAT 3';  R: 5'CGCTGAGTCTAAGTCGTCGTT 3' | Liver (12 h / 0 h) | 0.35 | 0.49 |
|  |  | Liver (36 h / 0 h) | 0.37 | 0.45 |
| ENSONIG00000016969  (Foxp3) | F: 5'CGCAGCCTCAGGTTACCACT 3';  R: 5'GGGAACCAAATCTGGGAGGAG 3' | Liver (12 h / 0 h) | 0.47 | 0.30 |
|  |  | Liver (36 h / 0 h) | 0.73 | 0.49 |
| ENSONIG00000014226  (Cathepsin La) | F: 5'GGAACCGACGACCAGAAATGC 3';  R: 5'TCATAGTAGATTCCTGACTGGTAA 3' | Liver (12 h / 0 h) | 2.71 | 2.25 |
|  |  | Liver (36 h / 0 h) | 1.48 | 1.54 |
| ENSONIG00000006671  (Cathepsin S) | F: 5'ACGGCTGCAATGGAGGCTTC 3';  R: 5'GCTTCAGAGCGTTCTCGTCC 3' | Gut (12 h / 0 h) | 1.40 | 1.61 |
|  |  | Gut (36 h / 0 h) | 1.39 | 1.13 |
| ENSONIG00000010953  (Ig heavy chain) | F: 5'CCACCGGCTTTAACCCTCCT 3';  R: 5'GCATTCCCAGCAGCGTGAGT 3' | Gut (12 h / 0 h) | 0.38 | 0.64 |
|  |  | Gut (36 h / 0 h) | 0.37 | 0.76 |
| ENSONIG00000017558  (Ig light chain) | F: 5'GTGGAGGAACCAGACTGAATGT 3'  R: 5'AGCTGATGCTGCCACTGCCAT 3' | Liver (12 h / 0 h) | 0.82 | 0.48 |
|  |  | Liver (36 h / 0 h) | 1.75 | 1.50 |
| ENSONIG00000011687  (haptoglobin) | F: 5'CAGAAGTTGCTGTGCTGGGTA 3'  R: 5'TTGGGCACAGTTCTGTTGGGT 3' | Liver (12 h / 0 h) | 1.51 | 1.05 |
|  |  | Liver (36 h / 0 h) | 1.51 | 1.20 |

Notes: FC is abbreviated for fold change.
