# Supplementary material for: Fish gut-liver immunity during homeostasis or inflammation revealed by integrative transcriptome and proteome studies
Source: Sci Rep. 2016 Nov 3;6:36048. doi: 10.1038/srep36048 (PMC5093735; doi:10.1038/srep36048)
Supplement: Supplementary Dataset 11 [file srep36048-s11.doc]

**Fish gut-liver immunity during homeostasis or inflammation revealed by integrative** **transcriptome and proteome studies**

Nan Wu^1^, Yu-Long Song^1,2^, Bei Wang^3^, Xiang-Yang Zhang^1,4^, Xu-Jie Zhang^1,5^, Ya-Li Wang^1^, Ying-Yin Cheng^1^, Dan-Dan Chen^1^, Xiao-Qin Xia^1^, Yi-Shan Lu^3^ & Yong-An Zhang^1,6^

^1^Institute of Hydrobiology, Chinese Academy of Sciences, Wuhan 430072, China

^2^Demorgen Bioinformation Technology Co. Ltd, Wuhan 430072, China

^3^Fisheries College, Guangdong Ocean University, Zhanjiang 524088, China

^4^University of Chinese Academy of Sciences, Beijing 100049, China

^5^College of Fisheries and Life Science, Shanghai Ocean University, Shanghai 201306, China

^6^State Key Laboratory of Freshwater Ecology and Biotechnology, Wuhan 430072, China

Correspondence and requests for materials should be addressed to Y.A.Z. (email: yzhang@ihb.ac.cn)

Supplementary data:

Dataset 1: Figures (S1-S5) & Tables (S1-S2) (Fig. S1 Involved GO terms and KEGG pathways of differential expressed transcripts between gut and liver; Fig. S2 Mostly matched immune related part of KEGG pathways by comparing gut and liver DGE data at different time points of either healthy or inflammational stages; Fig. S3 [Correlation](javascript:void(0);) [analysis](javascript:void(0);) among DGE or iTRAQ data of different time point; Fig. S4 Correlation analysis between fold changes of both qPCR and DGE data for validation; Fig. S5 Involved GO terms, KEGG pathways, and predicted protein domains of differential proteins between intestinal mucus and bile; Table S1 Quality of DGE data; Table S2 Detail information for qPCR validation);

Dataset 2: Table S3 All Identified Protein annot summary in iTRAQ result;

Dataset 3: Table S4 The Tilapia immune gene library, containing the detailed gene annotation and No. of genes in the second level

Dataset 4: Table S5 No. of differential immune transcripts between gut and liver;

Dataset 5: Table S6 No. of differential immune proteins between intestinal mucus and bile;

Dataset 6: Table S7 Differential immune transcripts between gut and liver in each region of the Venn diagram in Fig. 4A (the right one) comparing data of 0 h, 12 h, and 36 h;

Dataset 7: Table S8 Differential immune proteins between intestinal mucus and bile in each region of the Venn diagram in Fig. 5A (the right one) comparing data of 0 h and 36 h;

Dataset 8: Table S9 Common immune genes between liver advantage and bile advantage at 0h;

Dataset 9: Table S10 List of up or down regulated transcript in gut or liver comparing data of 0 h, 12 h, and 36 h;

Dataset 10: Table S11 List of up or down regulated proteins in mucus or bile comparing data of 0 h and 36 h.
